# Supplementary material for: Four New Sesquiterpenoids from the Rice Fermentation of Antrodiella albocinnamomea
Source: Molecules. 2022 May 23;27(10):3344. doi: 10.3390/molecules27103344 (PMC9147044; doi:10.3390/molecules27103344)
Supplement: Supplementary file 1 [file molecules-27-03344-s001.zip › molecules-1724049-supplementary.pdf]

# Four New Sesquiterpenoids from the Rice Fermentation of *Antrodiella albocinnamomea*

Min Guo [1], Ying-Zhong Liang [1], Xiu-Ming Cui [1, 2], Lin-Jiao Shao [1], Yin-Fei Li [1], and Xiao-Yan Yang [1, 2, *]

[1] Faculty of Life Science and Technology, Kunming University of Science and Technology, Kunming 650500, China, guomin@stu.kust.edu.cn (M. G.); lyz@stu.kust.edu.cn (Y.-Z. L.); 20120094@kust.edu.cn (X.-M. C.); shaolinjiao@stu.kust.edu.cn (L.-J. S.); liyinfei@stu.kust.edu.cn (Y.- F. L.); yangxy@kust.edu.cn (X.-Y. Y.)

[2] Yunnan Key Laboratory of Sustainable Utilization of *Panax Notoginseng*, Kunming 650500, China

[*]Correspondence: yangxy@kust.edu.cn (X.-Y. Y.); Tel.: +86-159-6946-8214 (X.-Y. Y.)

## Table of Contents

**Table S1.** Experimental and calculated $^{13}C$ data for possible structures of compounds **1**, **2** and **4** ($\delta$ in ppm)

**Table S2.** Regression analysis of $^{13}C$ NMR chemical displacement calculation and experimental value of eight possible configurations of compounds **1**, **2** and **4**

**Table S3.** DP4+ Analysis

**Figure S1.** Experimental and calculated ECD spectra of compound **1**

**Figure S2.** HR-ESI-MS spectrum of compound **1**

**Figure S3.** IR spectrum of compound **1**

**Figure S4.** $^{1}H$ NMR spectrum (600MHz, $CDCl_3$) of **1**

**Figure S5.** $^{13}C$ and DEPT spectrum (150MHz, $CDCl_3$) of **1**

**Figure S6.** HSQC spectrum of **1**

**Figure S7.** HMBC spectrum of **1**

**Figure S8.** $^{1}H$-$^{1}H$ COSY spectrum of **1**

**Figure S9.** ROESY spectrum of **1**

**Figure S10.** HR-ESI-MS spectrum of compound **2**

**Figure S11.** IR spectrum of compound **2**

**Figure S12.** $^{1}H$ NMR spectrum (600MHz, $CDCl_3$) of **2**

**Figure S13.** $^{13}C$ and DEPT spectrum (150MHz, $CDCl_3$) of **2**

**Figure S14.** HSQC spectrum of **2**

**Figure S15.** HMBC spectrum of **2**

**Figure S16.** $^{1}H$-$^{1}H$ COSY spectrum of **2**

**Figure S17.** ROESY spectrum of **2**

**Figure S18.** HR-ESI-MS spectrum of compound **3**

**Figure S19.** IR spectrum of compound **3**

**Figure S20.** $^{1}H$ NMR spectrum (600MHz, $CDCl_3$) of **3**

**Figure S21.** $^{13}C$ and DEPT spectrum (150MHz, $CDCl_3$) of **3**

**Figure S22.** HSQC spectrum of **3**

**Figure S23.** HMBC spectrum of **3**

**Figure S24.** $^1$H-$^1$H COSY spectrum of **3**

**Figure S25.** ROESY spectrum of **3**

**Figure S26.** Experimental and calculated ECD spectra of compound **4**

**Figure S27.** HR-ESI-MS spectrum of compound **4**

**Figure S28.** IR spectrum of compound **4**

**Figure S29.** $^1$H NMR spectrum (600MHz, $CD_3OD$) of **4**

**Figure S30.** $^{13}$C and DEPT spectrum (150MHz, $CD_3OD$) of **4**

**Figure S31.** HSQC spectrum of **4**

**Figure S32.** HMBC spectrum of **4**

**Figure S33.** $^1$H-$^1$H COSY spectrum of **4**

**Figure S34.** ROESY spectrum of **4**

**Figure S35.** There are possible configurations of compounds **1**, **2** and **4**

**Figure S36.** Linear correlation plots of calculate-experimental $^{13}$C NMR chemical shift values for (6*R*,7*S*)-**1a**; (6*R*,7*R*)-**1b**; (6*S*,7*R*)-**1c**; (6*S*,7*S*)-**1d**; (5*S*,6*S*)-**2a**; (5*S*,6*R*)-**2b**; (5*R*,6*S*)-**2c**; (5*R*,6*R*)-**2d**, (5*R*,9*S*,13*S*)-**4a**; (5*R*,9*S*,13*R*)-**4b**; (5*S*,9*S*,13*S*)-**4c**; (5*R*,9*R*,13*S*)-**4d**; (5*S*,9*R*,13*R*)-**4e**; (5*S*,9*R*,13*S*)-**4f**; (5*R*,9*R*,13*R*)-**4g**; (5*S*,9*S*,13*R*)-**4h**

**Figure S37.** Antibacterial test of compounds **2** and **6**

**Table S4.** The Boltzmann distribution proportion of its dominant conformation of the compound **2** (5*S*,6*S*)

**Table S5.** Compound **3** (*S*) within 3.0 kcal/mol, and the Boltzmann distribution proportion of its dominant conformation

**Figure S38.** Experimental optical rotation of compound **2**

**Figure S39.** Experimental optical rotation of compound **3**

**Table S1** Experimental and calculated $^{13}C$ data for possible structures of compounds **1**, **2** and **4** ($\delta$ in ppm)

| NO. | $\delta_C$, Exptl[A] | **1** | | | | | | | |
|---|---|---|---|---|---|---|---|---|---|
| | | **1a** | | **1b** | | **1c** | | **1d** | |
| | | $\delta_C$, calcd[B] | $\delta_C$, $\Delta\delta$[C] | $\delta_C$, calcd[B] | $\delta_C$, $\Delta\delta$[C] | $\delta_C$, calcd[B] | $\delta_C$, $\Delta\delta$[C] | $\delta_C$, calcd[B] | $\delta_C$, $\Delta\delta$[C] |
| 10 | 38.8 | 38.3 | -0.5 | 38.6 | -0.2 | 37.9 | -0.9 | 38.6 | -0.2 |
| 9 | 35.7 | 34.9 | -0.8 | 34.8 | -0.9 | 34.7 | -1.0 | 34.8 | -0.9 |
| 8 | 213.1 | 211.8 | -1.3 | 211.5 | -1.6 | 211.8 | -1.3 | 211.4 | -1.7 |
| 7 | 91.5 | 77.7 | -13.8 | 79.0 | -12.5 | 77.6 | -13.9 | 78.9 | -12.6 |
| 6 | 45.5 | 56.8 | 11.3 | 56.3 | 10.8 | 56.7 | 11.2 | 56.6 | 11.1 |
| 11 | 55.5 | 37.8 | -17.7 | 37.6 | -17.9 | 37.9 | -17.6 | 37.6 | -17.9 |
| 1 | 32.3 | 39.7 | 7.4 | 41.1 | 8.8 | 39.8 | 7.5 | 41.1 | 8.8 |
| 2 | 194.7 | 190.4 | -4.3 | 188.5 | -6.2 | 190.7 | -4.0 | 188.4 | -6.3 |
| 3 | 145 | 140.6 | -4.4 | 141.6 | -3.4 | 140.6 | -4.4 | 141.7 | -3.3 |
| 4 | 121.3 | 112.9 | -8.4 | 105.9 | -15.4 | 112.8 | -8.5 | 105.7 | -15.6 |
| 5 | 25.2 | 28.1 | 2.9 | 28.9 | 3.7 | 28.2 | 3.0 | 28.9 | 3.7 |
| 12 | 24.3 | 25.5 | 1.2 | 30.9 | 6.6 | 34.2 | 9.9 | 26.1 | 1.8 |
| 13 | 24.4 | 34.0 | 9.6 | 26.1 | 1.7 | 25.6 | 1.2 | 30.7 | 6.3 |
| 14 | 26.8 | 25.4 | -1.4 | 26.9 | 0.1 | 25.3 | -1.5 | 27.1 | 0.3 |

| NO. | $\delta_C$, exptl[A] | **2** | | | | | | | |
|---|---|---|---|---|---|---|---|---|---|
| | | **2a** | | **2b** | | **2c** | | **2d** | |
| | | $\delta_C$, calcd[B] | $\delta_C$, $\Delta\delta$[C] | $\delta_C$, calcd[B] | $\delta_C$, $\Delta\delta$[C] | $\delta_C$, calcd[B] | $\delta_C$, $\Delta\delta$[C] | $\delta_C$, calcd[B] | $\delta_C$, $\Delta\delta$[C] |
| 9 | 36.5 | 37.0 | 0.5 | 36.5 | 0.00 | 36.8 | 0.3 | 37.0 | 0.5 |
| 8 | 33.8 | 34.6 | 0.8 | 34.3 | 0.51 | 34.3 | 0.5 | 34.6 | 0.8 |
| 7 | 213.4 | 220.4 | 7.0 | 221.2 | 7.80 | 221.3 | 7.9 | 220.4 | 7.0 |
| 6 | 80 | 79.8 | -0.2 | 79.7 | -0.34 | 79.5 | -0.5 | 79.8 | -0.2 |
| 5 | 59.7 | 61.5 | 1.8 | 61.8 | 2.09 | 61.6 | 1.9 | 61.5 | 1.8 |
| 10 | 37 | 37.2 | 0.2 | 36.8 | -0.19 | 37.0 | 0.0 | 37.2 | 0.2 |
| 4 | 37.6 | 41.4 | 3.8 | 42.1 | 4.54 | 42.0 | 4.4 | 41.4 | 3.8 |
| 3 | 150.8 | 154.7 | 3.9 | 155.2 | 4.43 | 155.3 | 4.5 | 154.7 | 3.9 |
| 2 | 146.4 | 146.9 | 0.5 | 146.6 | 0.25 | 146.6 | 0.2 | 146.9 | 0.5 |
| 1 | 34.3 | 36.4 | 2.1 | 36.4 | 2.05 | 36.0 | 1.7 | 36.4 | 2.1 |
| 12 | 24.7 | 27.2 | 2.5 | 27.1 | 2.38 | 24.6 | -0.1 | 27.2 | 2.5 |
| 11 | 26.8 | 25.0 | -1.8 | 24.4 | -2.39 | 27.4 | 0.6 | 25.0 | -1.8 |
| 14 | 189.5 | 188.8 | -0.7 | 188.9 | -0.61 | 188.8 | -0.7 | 188.8 | -0.7 |
| 13 | 25.2 | 25.8 | 0.6 | 26.2 | 1.03 | 26.3 | 1.1 | 25.8 | 0.6 |

[A]Recorded in $CDCl_3$ at 150Hz. [B]Calculated in chloroform. [C]$\Delta\delta=\delta$calcd-$\delta$exptl

| NO. | $\delta_C$, Exptl[A] | **4** | | | |
|---|---|---|---|---|---|
| | | **4a** | **4b** | **4c** | **4d** |

| | | δC, calcd[B] | δC, Δδ[C] | δC, calcd[B] | δC, Δδ[C] | δC, calcd[B] | δC, Δδ[C] | δC, calcd[B] | δC, Δδ[C] |
|---|---|---|---|---|---|---|---|---|---|
| 6 | 207.4 | 208.8 | 1.4 | 209.9 | 2.5 | 203.9 | -3.5 | 206.0 | -1.4 |
| 5 | 50.6 | 48.5 | -2.1 | 46.3 | -4.3 | 48.2 | -2.4 | 50.3 | -0.3 |
| 4 | 41.2 | 42.3 | 1.1 | 45.4 | 4.2 | 52.1 | 10.9 | 43.3 | 2.1 |
| 9 | 78.6 | 78.8 | 0.2 | 78.3 | -0.3 | 73.8 | -4.8 | 74.3 | -4.3 |
| 8 | 165 | 168.1 | 3.1 | 169.4 | 4.4 | 171.3 | 6.3 | 171.3 | 6.3 |
| 7 | 131.5 | 129.5 | -2.0 | 129.2 | -2.3 | 131.1 | -0.4 | 130.5 | -1.0 |
| 3 | 59.1 | 56.0 | -3.1 | 55.6 | -3.5 | 55.0 | -4.1 | 54.0 | -5.1 |
| 2 | 38.9 | 37.4 | -1.5 | 37.6 | -1.3 | 37.2 | -1.7 | 37.3 | -1.6 |
| 1 | 47.4 | 45.2 | -2.2 | 45.4 | -2.0 | 45.7 | -1.7 | 45.4 | -2.0 |
| 15 | 13.2 | 14.4 | 1.2 | 14.4 | 1.2 | 14.6 | 1.4 | 14.5 | 1.3 |
| 13 | 75.5 | 73.2 | -2.3 | 72.1 | -3.4 | 76.2 | 0.7 | 74.9 | -0.6 |
| 14 | 18 | 16.7 | -1.3 | 16.5 | -1.5 | 20.3 | 2.3 | 16.2 | -1.8 |
| 12 | 26.1 | 24.2 | -1.9 | 18.7 | -7.4 | 25.5 | -0.6 | 26.2 | 0.1 |
| 10 | 32.1 | 31.4 | -0.7 | 31.1 | -1.0 | 28.8 | -3.3 | 28.8 | -3.3 |
| 11 | 31.9 | 31.4 | -0.5 | 31.0 | -0.9 | 29.2 | -2.7 | 29.0 | -2.9 |

| NO. | δC, exptl[A] | **4e** | | **4f** | | **4g** | | **4h** | |
|---|---|---|---|---|---|---|---|---|---|
| | | δC, calcd[B] | δC, Δδ[C] | δC, calcd[B] | δC, Δδ[C] | δC, calcd[B] | δC, Δδ[C] | δC, calcd[B] | δC, Δδ[C] |
| 6 | 207.4 | 208.9 | 1.5 | 209.8 | 2.4 | 203.9 | -3.5 | 206.2 | -1.2 |
| 5 | 50.6 | 48.7 | -1.9 | 46.4 | -4.2 | 48.5 | -2.1 | 50.1 | -0.5 |
| 4 | 41.2 | 42.1 | 0.9 | 45.2 | 4.0 | 51.7 | 10.5 | 43.3 | 2.1 |
| 9 | 78.6 | 78.8 | 0.2 | 78.5 | -0.1 | 73.9 | -4.7 | 74.2 | -4.4 |
| 8 | 165 | 168.1 | 3.1 | 169.4 | 4.4 | 171.2 | 6.2 | 171.5 | 6.5 |
| 7 | 131.5 | 129.6 | -1.9 | 129.0 | -2.5 | 131.3 | -0.2 | 130.5 | -1.0 |
| 3 | 59.1 | 56.1 | -3.0 | 55.6 | -3.5 | 55.1 | -4.0 | 54.2 | -4.9 |
| 2 | 38.9 | 37.5 | -1.4 | 37.7 | -1.2 | 37.4 | -1.5 | 37.4 | -1.5 |
| 1 | 47.4 | 45.2 | -2.2 | 45.6 | -1.8 | 45.3 | -2.1 | 45.5 | -1.9 |
| 15 | 13.2 | 14.3 | 1.1 | 14.4 | 1.2 | 14.6 | 1.4 | 14.6 | 1.4 |
| 13 | 75.5 | 73.2 | -2.3 | 72.3 | -3.2 | 76.3 | 0.8 | 74.8 | -0.7 |
| 14 | 18 | 16.9 | -1.1 | 16.2 | -1.8 | 20.3 | 2.3 | 16.3 | -1.7 |
| 12 | 26.1 | 24.3 | -1.8 | 18.8 | -7.3 | 25.5 | -0.6 | 26.2 | 0.1 |
| 10 | 32.1 | 31.4 | -0.7 | 31.6 | -0.5 | 29.1 | -3.0 | 29.1 | -3.0 |
| 11 | 31.9 | 31.4 | -0.5 | 31.4 | -0.5 | 29.2 | -2.7 | 29.0 | -2.9 |

[A]Recorded in $CD_3OD$ at 150Hz. [B]Calculated in methanol. [C]Δ*δ*=*δ*calcd-*δ*exptl

**Table S2** Regression analysis of $^{13}C$ NMR chemical displacement calculation and experimental value of eight possible configurations of compounds **1**, **2** and **4**

| No. | | configuration | $R^2$ | RMSE | DP4+ possibility (%) |
|---|---|---|---|---|---|
| **1** | **1a** | 6*R*,7*S* | 0.9855 | 8.01 | 56.02 |
| | **1b** | 6*R*,7*R* | 0.9842 | 8.54 | 8.65 |
| | **1c** | 6*S*,7*R* | 0.9854 | 8.03 | 28.61 |
| | **1d** | 6*S*,7*S* | 0.9841 | 8.59 | 6.71 |
| **2** | **2a** | 5*S*,6*S* | 0.9991 | 2.63 | 73.72 |
| | **2b** | 5*S*,6*R* | 0.9988 | 2.96 | 0.02 |
| | **2c** | 5*R*,6*S* | 0.9990 | 2.81 | 15.94 |
| | **2d** | 5*R*,6*R* | 0.9991 | 2.64 | 10.32 |
| **4** | **4a** | 5*R*,9*S*,13*S* | 0.9993 | 1.83 | 30.72 |
| | **4b** | 5*R*,9*S*,13*R* | 0.9975 | 3.24 | 0.00 |
| | **4c** | 5*S*,9*S*,13*S* | 0.9946 | 4.07 | 0.00 |
| | **4d** | 5*R*,9*R*,13*S* | 0.9978 | 2.87 | 0.00 |
| | **4e** | 5*S*,9*R*,13*R* | 0.9993 | 1.79 | 69.28 |
| | **4f** | 5*S*,9*R*,13*S* | 0.9976 | 3.18 | 0.00 |
| | **4g** | 5*R*,9*R*,13*R* | 0.9948 | 3.97 | 0.00 |
| | **4h** | 5*S*,9*S*,13*R* | 0.9978 | 2.85 | 0.00 |

**Table S3** DP4+ Analysis

| No. | **1** (%) | | | | **2** (%) | | | |
|---|---|---|---|---|---|---|---|---|
| | **1a** | **1b** | **1c** | **1d** | **2a** | **2b** | **2c** | **2d** |
| sDP4+ (H data) | - | - | - | - | - | - | - | - |
| sDP4+ (C data) | 56.02 | 8.65 | 28.61 | 6.71 | 40.66 | 0.67 | 34.53 | 24.14 |
| sDP4+ (all data) | 56.02 | 8.65 | 28.61 | 6.71 | 40.66 | 0.67 | 34.53 | 24.14 |
| uDP4+ (H data) | - | - | - | - | - | - | - | - |
| uDP4+ (C data) | - | - | - | - | 66.22 | 1.32 | 16.85 | 15.61 |
| uDP4+ (all data) | - | - | - | - | 66.22 | 1.32 | 16.85 | 15.61 |
| DP4+ (H data) | - | - | - | - | - | - | - | - |
| DP4+ (C data) | - | - | - | - | 73.72 | 0.02 | 15.94 | 10.32 |
| DP4+ (all data) | - | - | - | - | 73.72 | 0.02 | 15.94 | 10.32 |

| No. | **4** (%) | | | | | | | |
|---|---|---|---|---|---|---|---|---|
| | **4a** | **4b** | **4c** | **4d** | **4e** | **4f** | **4g** | **4h** |
| sDP4+ (H data) | - | - | - | - | - | - | - | - |
| sDP4+ (C data) | 39.36 | 0.00 | 0.00 | 0.00 | 60.63 | 0.00 | 0.00 | 0.00 |
| sDP4+ (all data) | 39.36 | 0.00 | 0.00 | 0.00 | 60.63 | 0.00 | 0.00 | 0.00 |
| uDP4+ (H | - | - | - | - | - | - | - | - |

| | | | | | | | | |
|---|---|---|---|---|---|---|---|---|
| data) | | | | | | | | |
| uDP4+ (C data) | 40.53 | 0.01 | 0.00 | 0.04 | 59.34 | 0.03 | 0.00 | 0.05 |
| uDP4+ (all data) | 40.53 | 0.01 | 0.00 | 0.04 | 59.34 | 0.03 | 0.00 | 0.05 |
| DP4+ (H data) | - | - | - | - | - | - | - | - |
| DP4+ (C data) | 30.72 | 0.00 | 0.00 | 0.00 | 69.28 | 0.00 | 0.00 | 0.00 |
| DP4+ (all data) | 30.72 | 0.00 | 0.00 | 0.00 | 69.28 | 0.00 | 0.00 | 0.00 |

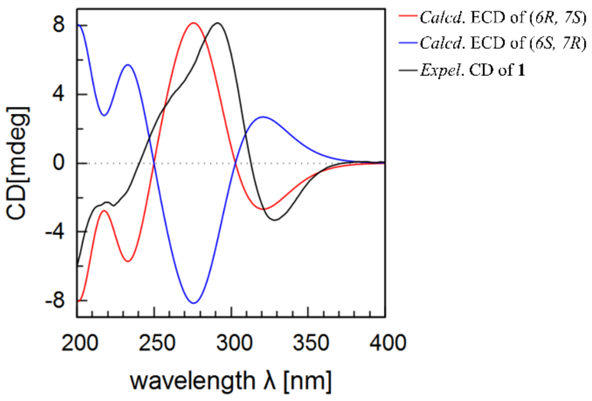


**Figure S1.** Experimental and calculated ECD spectra of compound **1**

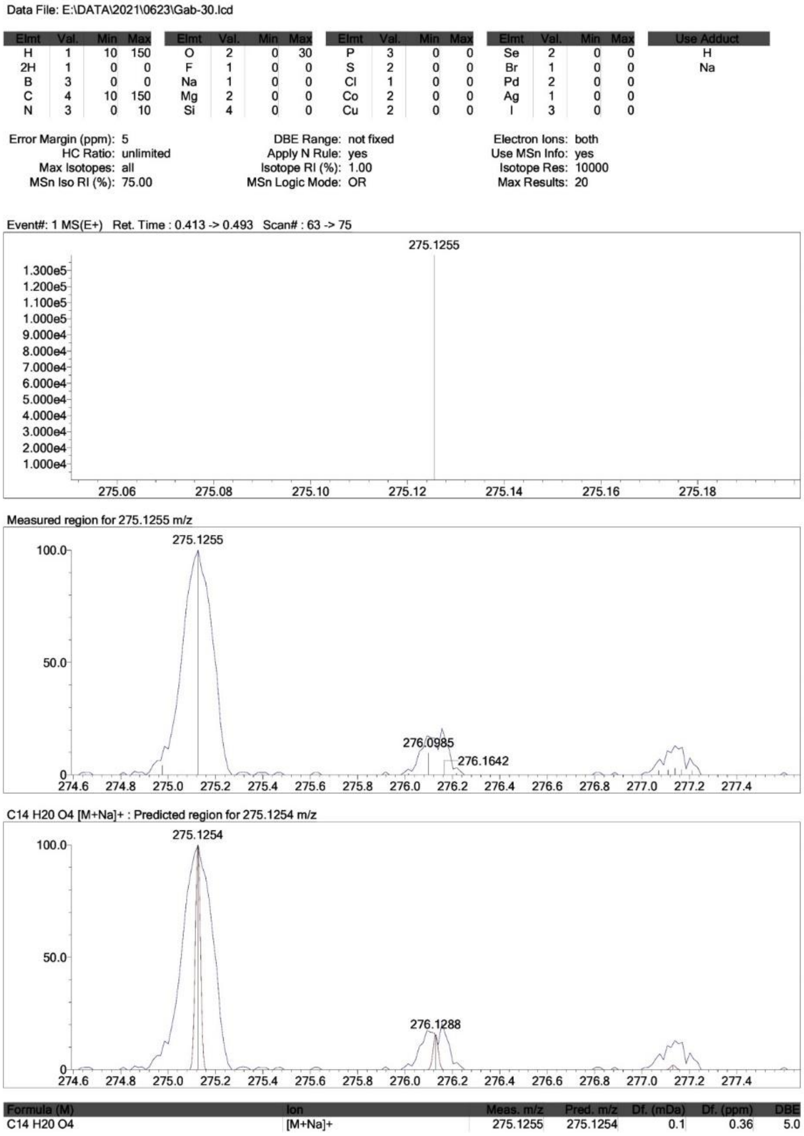


**Figure S2.** HR-ESI-MS spectrum of compound **1**

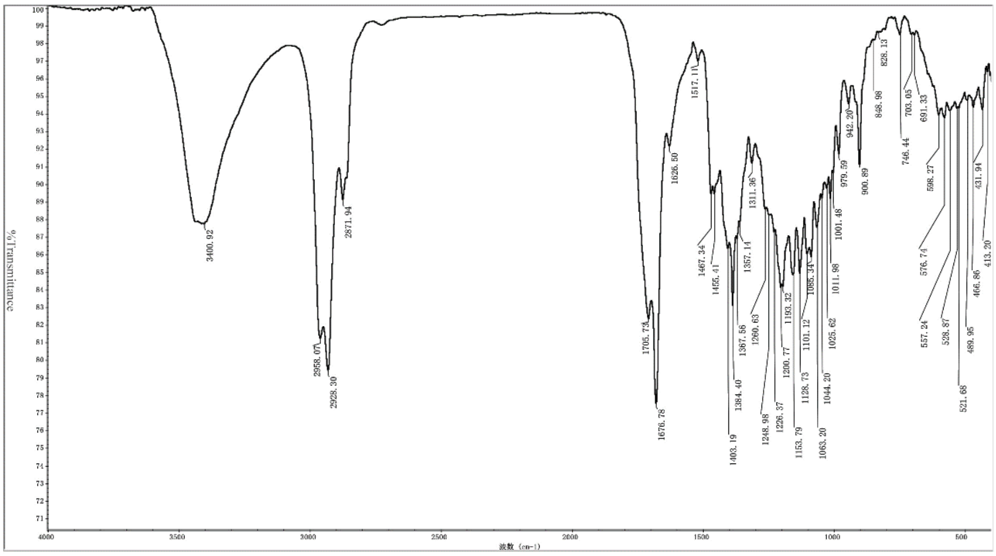


Sample Name: Gab-30
KBr Pellets
Date: 02/07/2021 13:23:51 (GMT+08:00)
Instrument model: NICOLET iS10
Software version: OMNIC 9.8.372

Sample Scans: 16
Background Scans: 16
resolving Power: 4.000
Sampling gain: 1.0
Moving Mirror Speed: 0.4747
Diaphragm: 80.00

**Figure S3.** IR spectrum of compound **1**

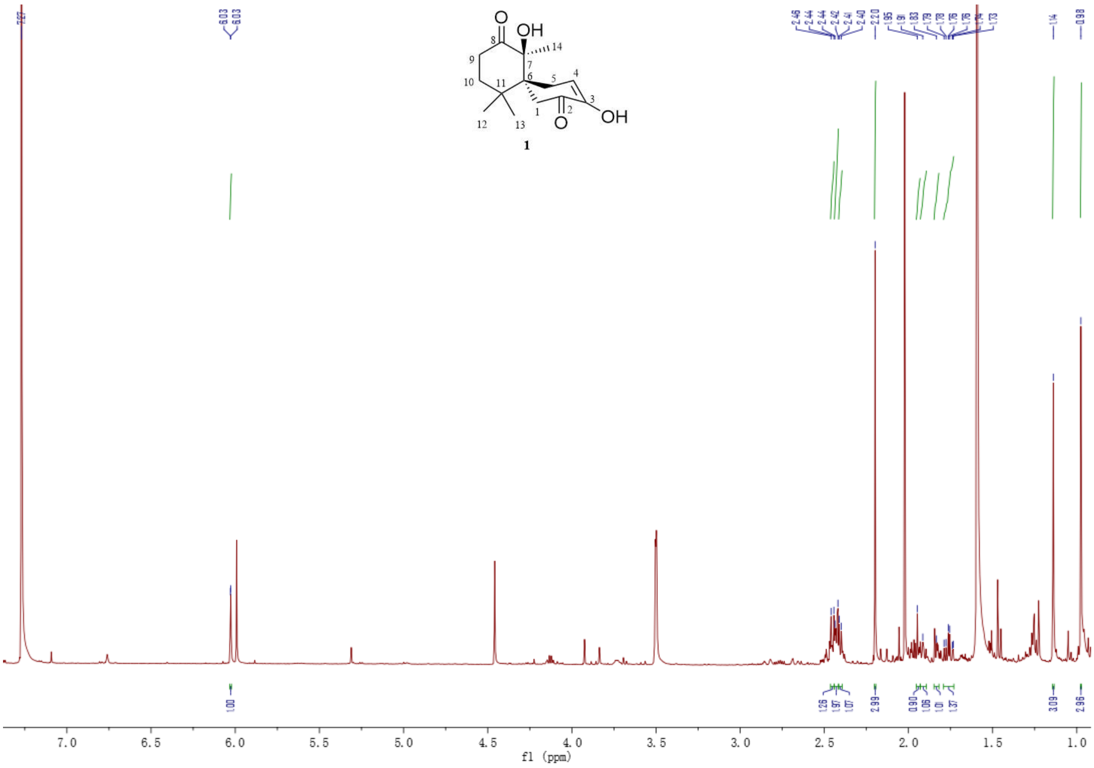


**Figure S4.** $^{1}$H NMR spectrum (600MHz, $CDCl_3$) of **1**

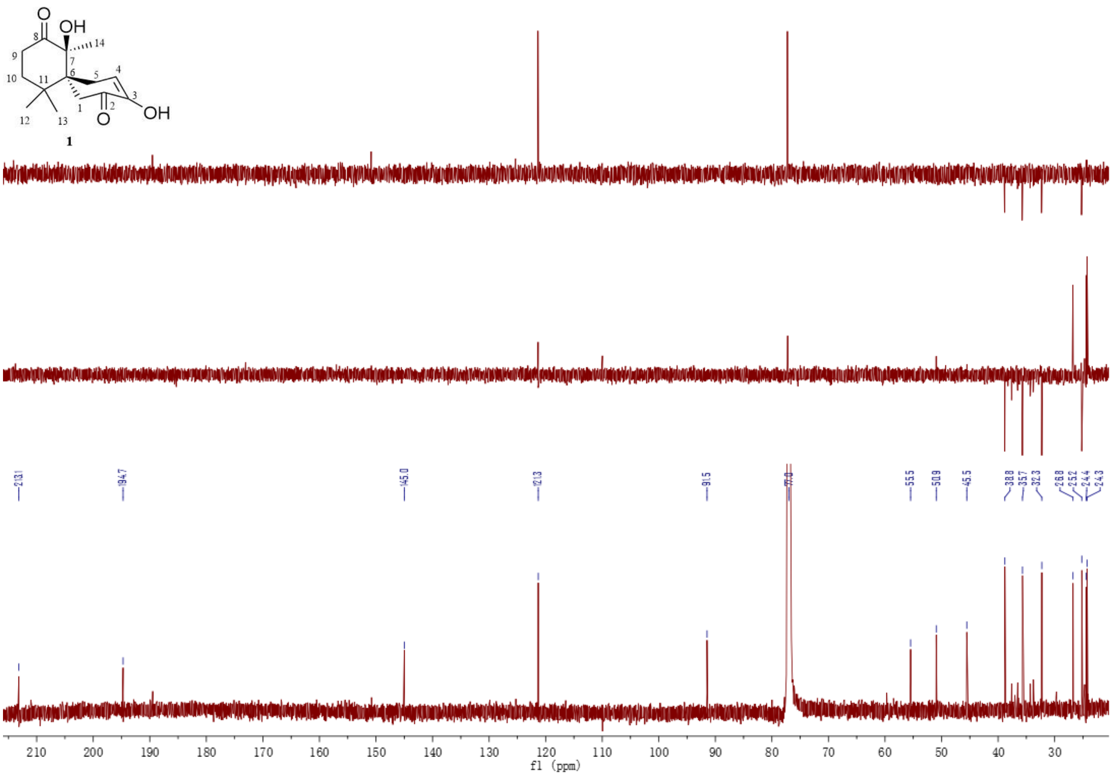


**Figure S5.** $^{13}C$ and DEPT spectrum (150MHz, $CDCl_3$) of **1**

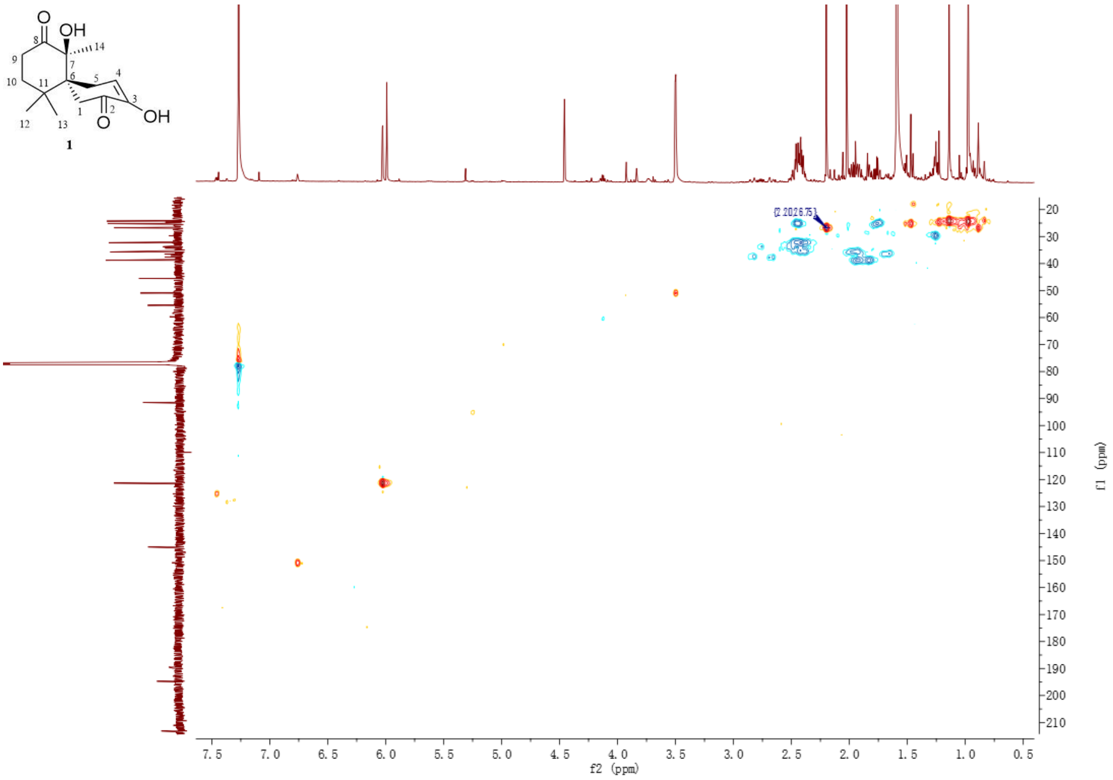


**Figure S6.** HSQC spectrum of **1**

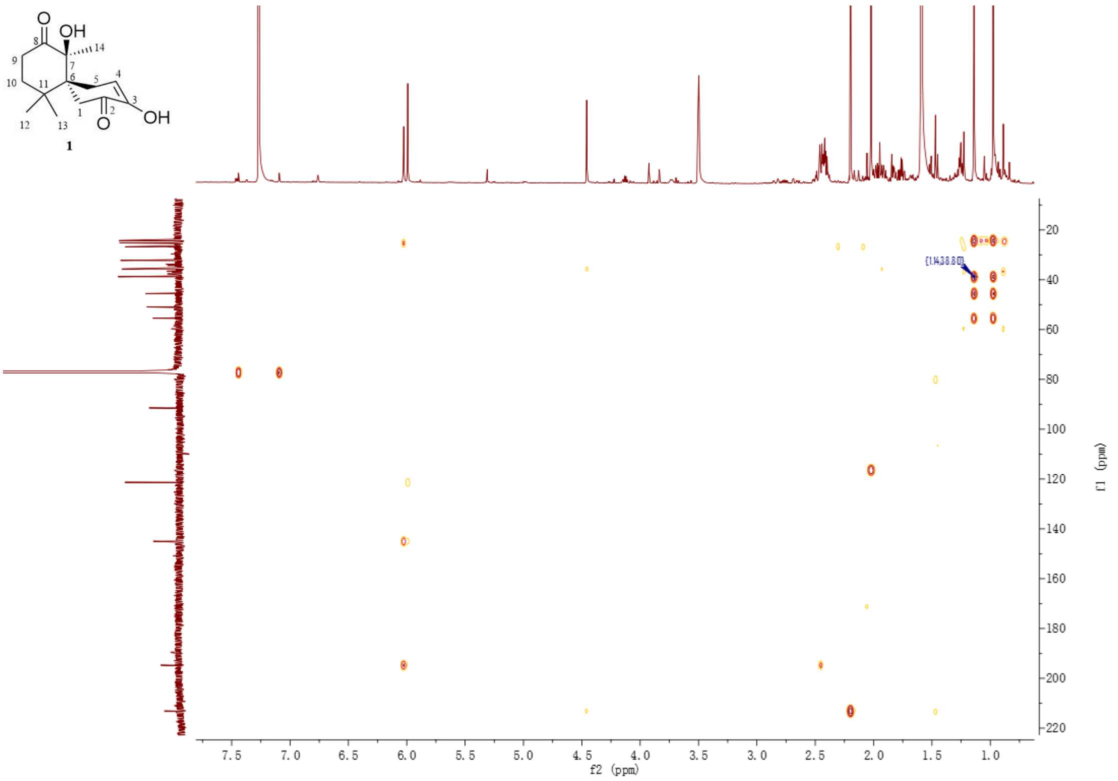


**Figure S7.** HMBC spectrum of **1**

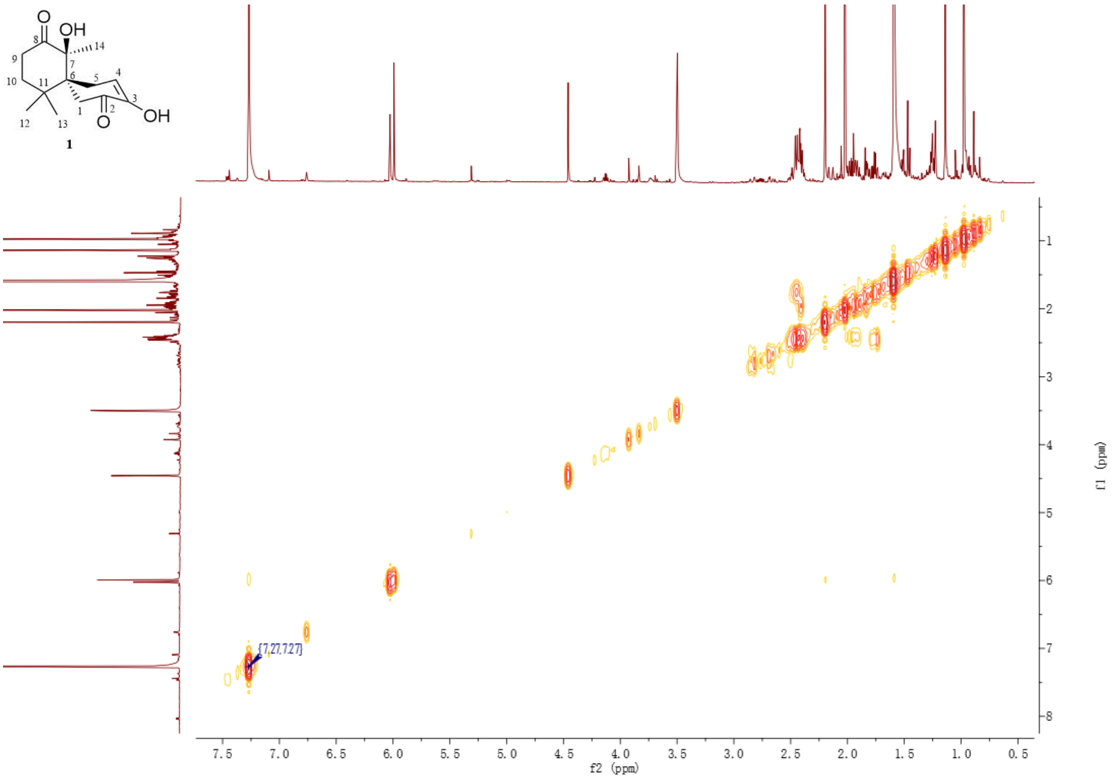


**Figure S8.** $^{1}H$-$^{1}H$ COSY spectrum of **1**

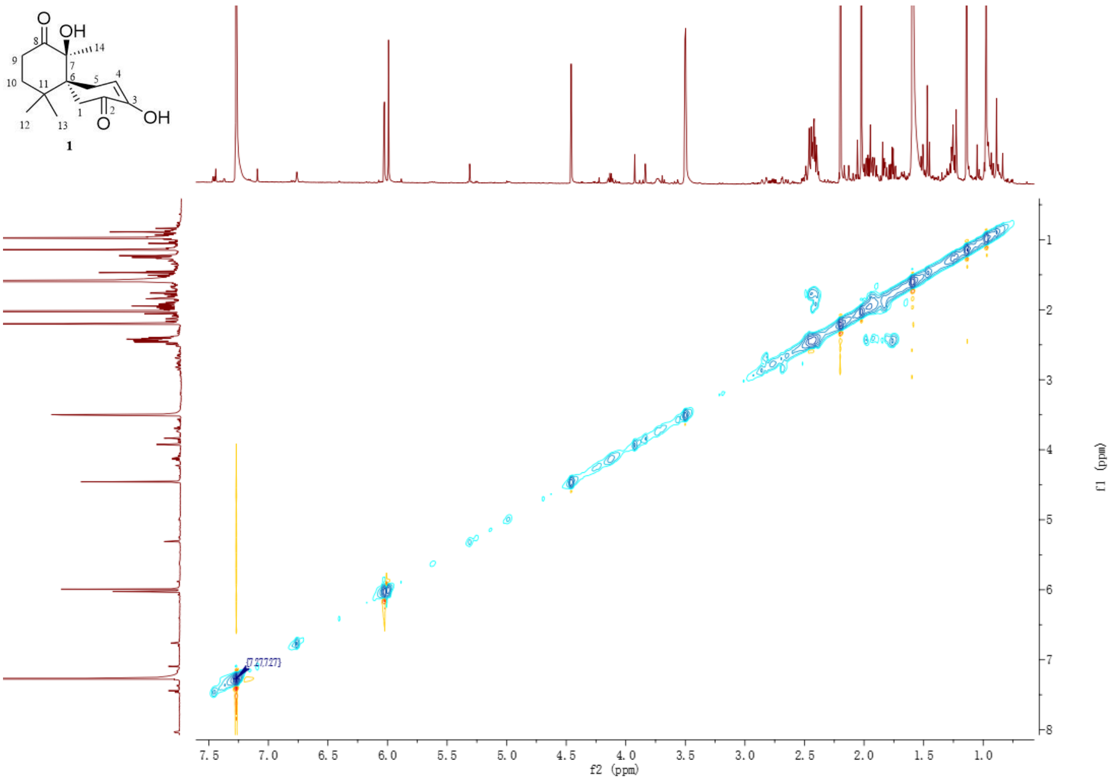


**Figure S9.** ROESY spectrum of **1**

**Elemental Composition Report** **Page 1**

**Single Mass Analysis**
Tolerance = 10.0 PPM / DBE: min = -10.0, max = 120.0
Selected filters: None

Monoisotopic Mass, Odd and Even Electron Ions
18 formula(e) evaluated with 1 results within limits (up to 51 closest results for each mass)
Elements Used:
C: 0-200 H: 0-400 O: 1-4

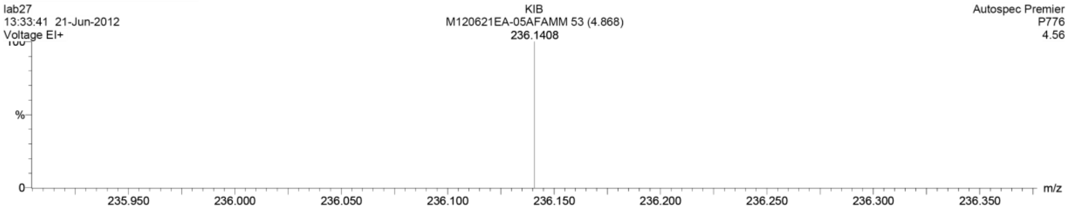


| | | | | | | |
|---|---|---|---|---|---|---|
| Minimum: | | | | -10.0 | | |
| Maximum: | | 100.0 | 10.0 | 120.0 | | |
| Mass | Calc. Mass | mDa | PPM | DBE | i-FIT | Formula |
| 236.1408 | 236.1412 | -0.4 | -1.7 | 5.0 | 5546025.5 | C14 H20 O3 |

**Figure S10.** HR-ESI-MS spectrum of compound **2**

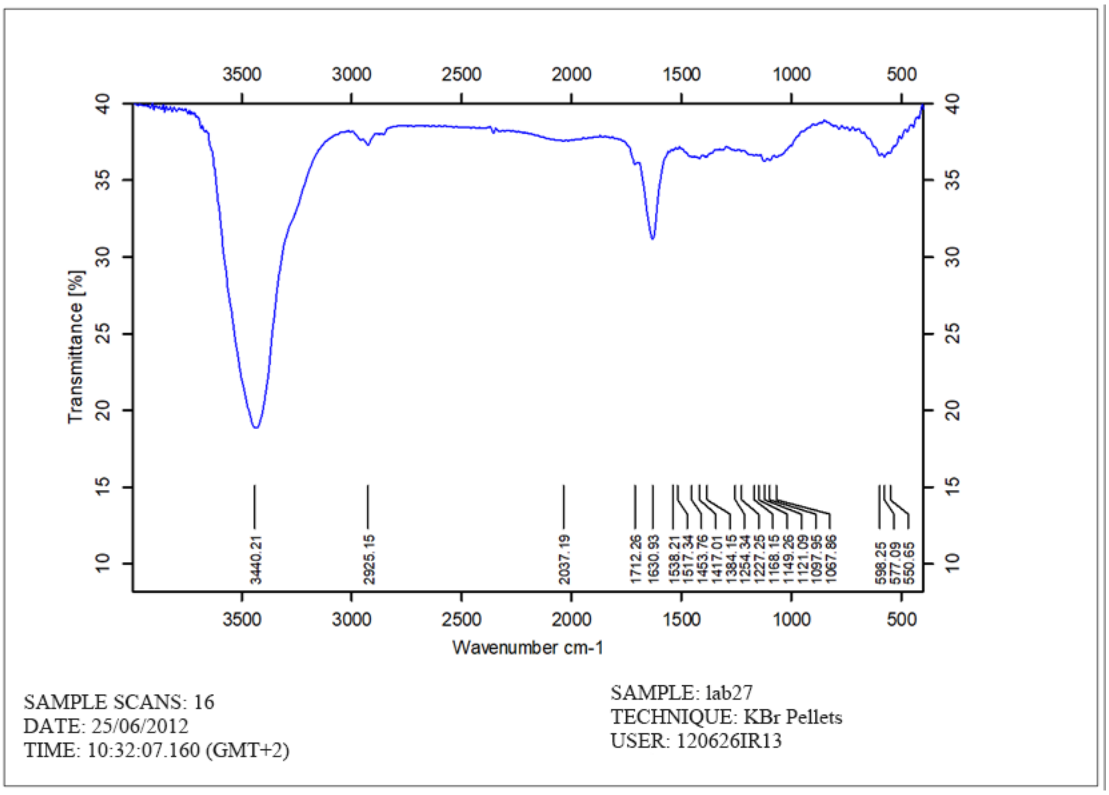


**Figure S11.** IR spectrum of compound **2**

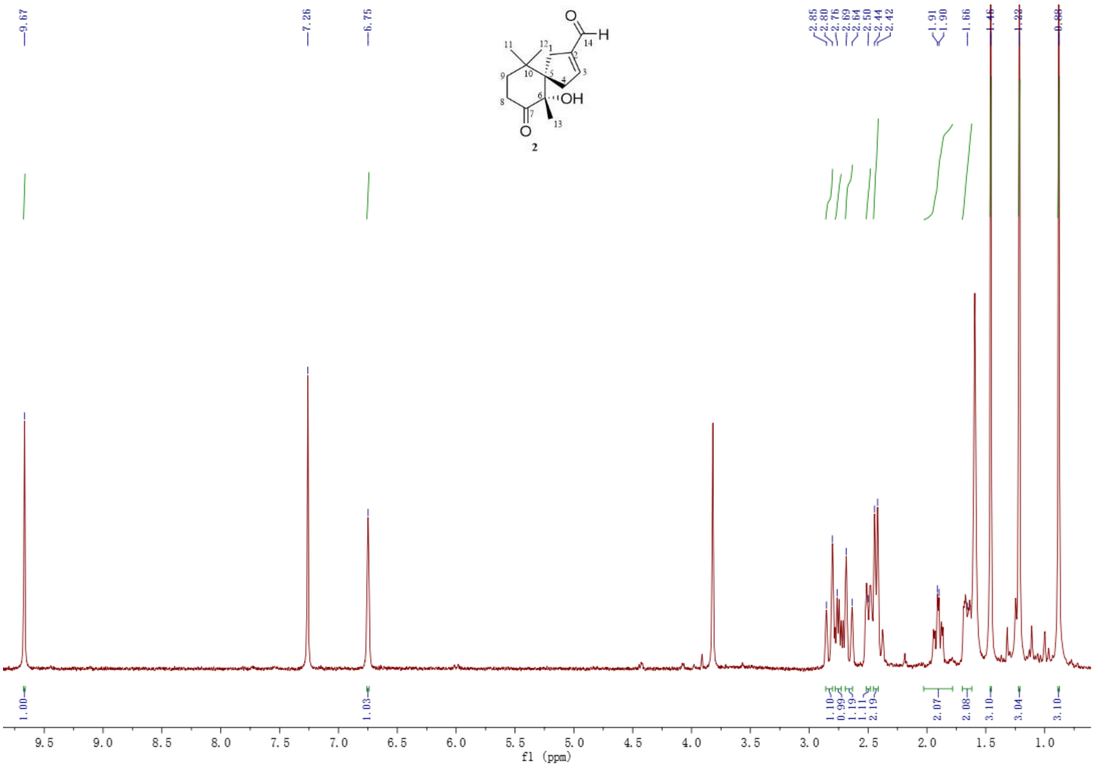


**Figure S12.** $^1H$ NMR spectrum (600MHz, $CDCl_3$) of **2**

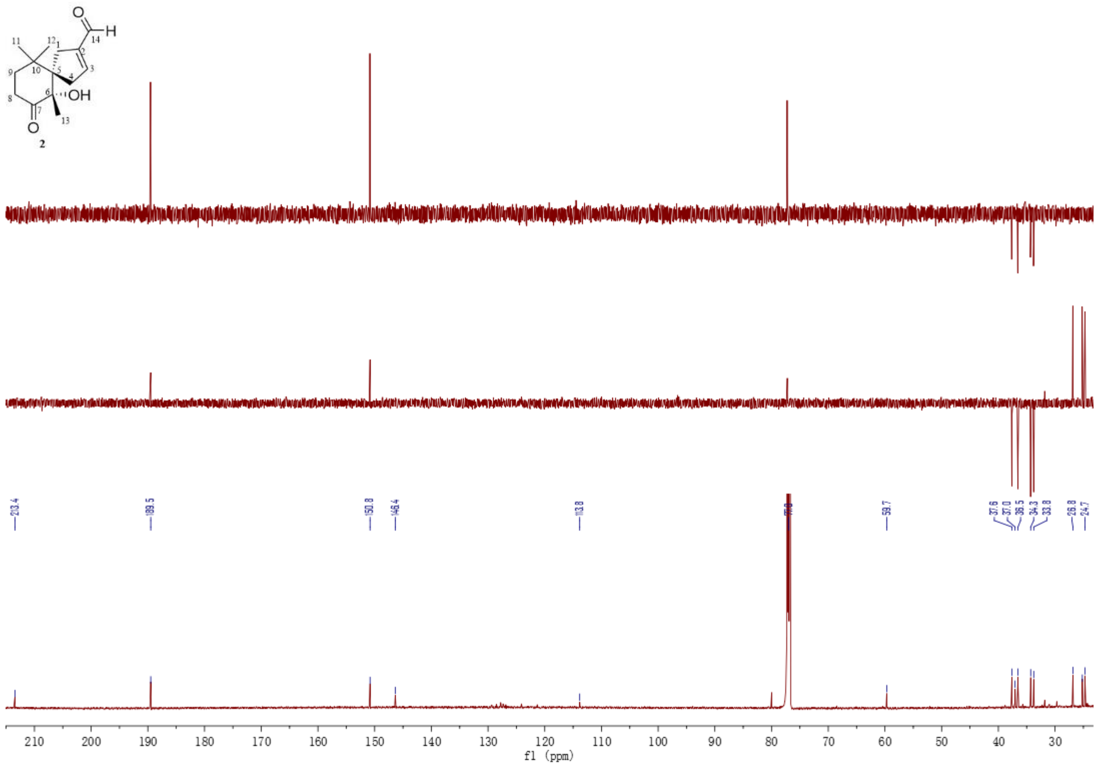

**Figure S13.** $^{13}C$ and DEPT spectrum (150MHz, $CDCl_3$) of **2**

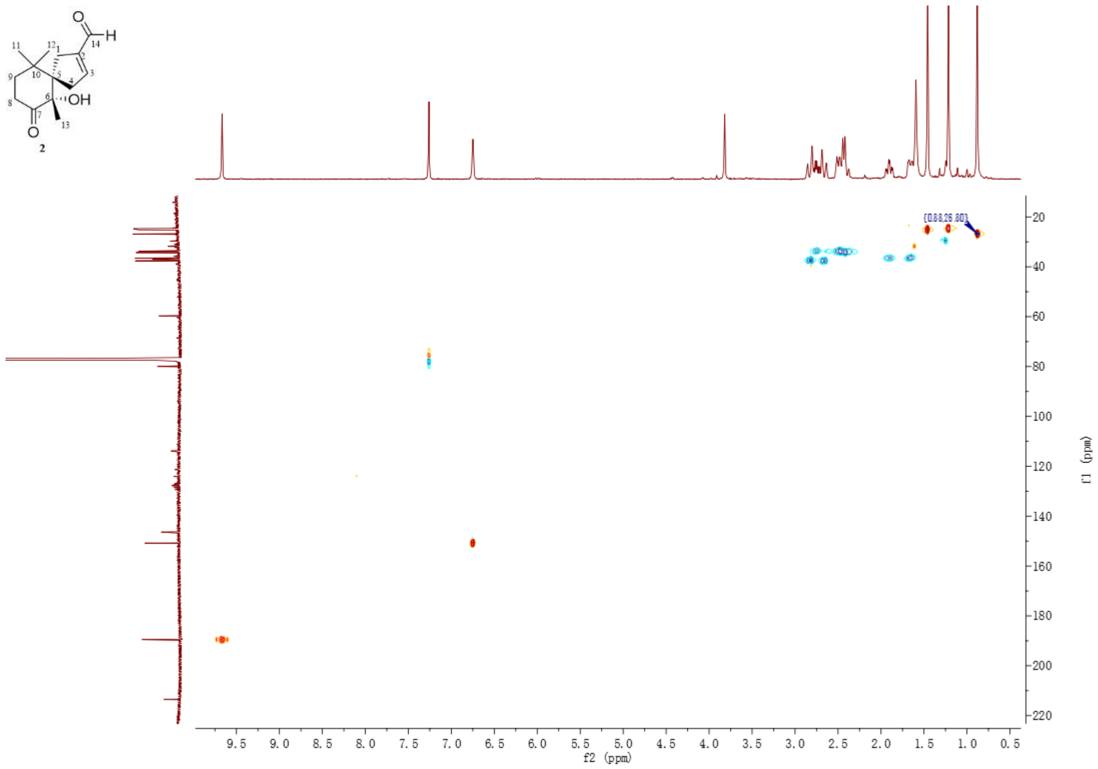

**Figure S14.** HSQC spectrum of **2**

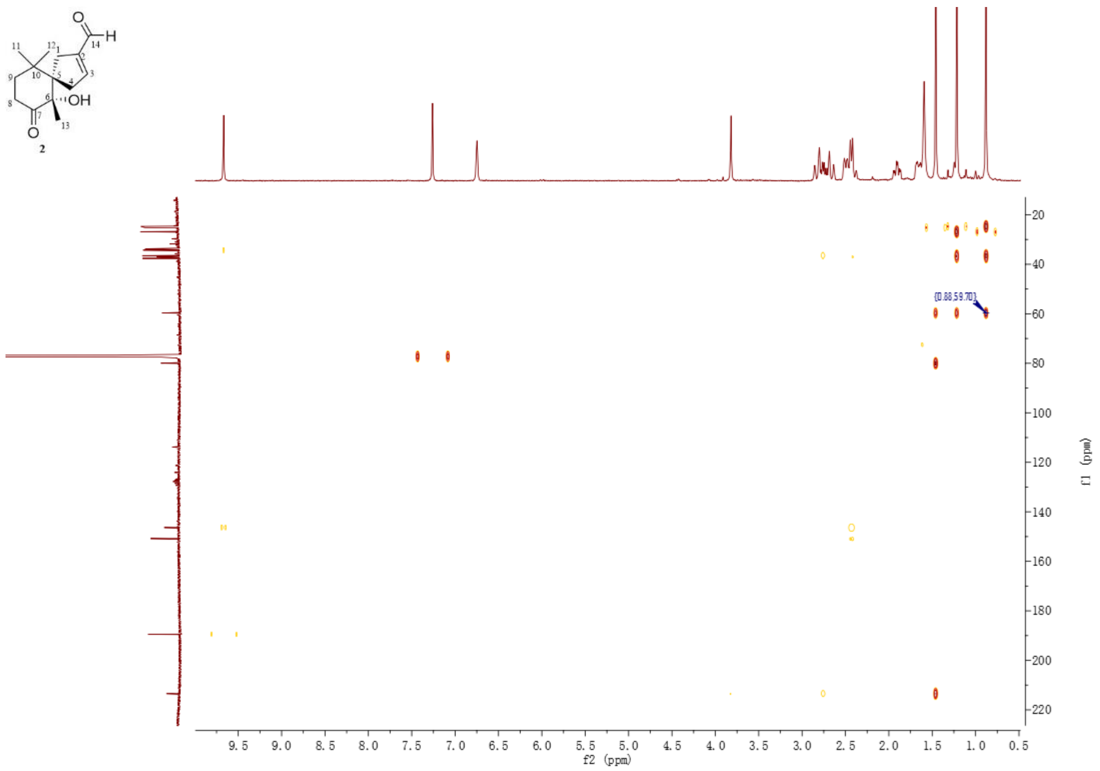


**Figure S15.** HMBC spectrum of **2**

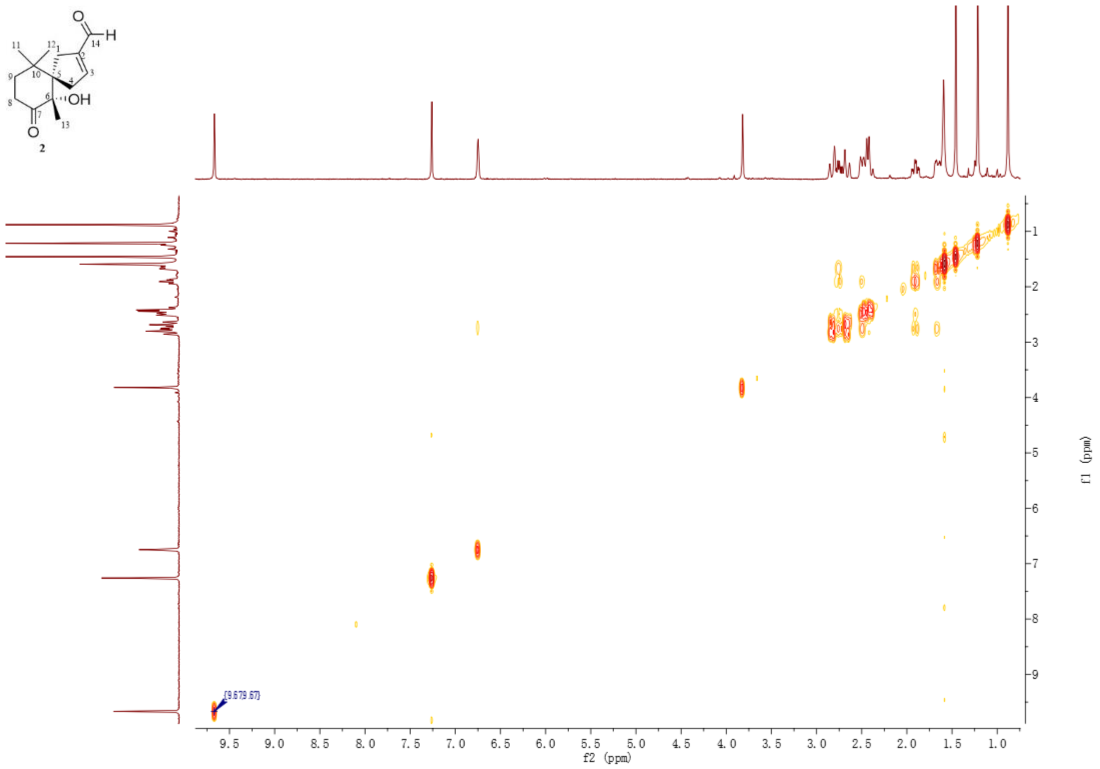


**Figure S16.** $^{1}H$-$^{1}H$ COSY spectrum of **2**

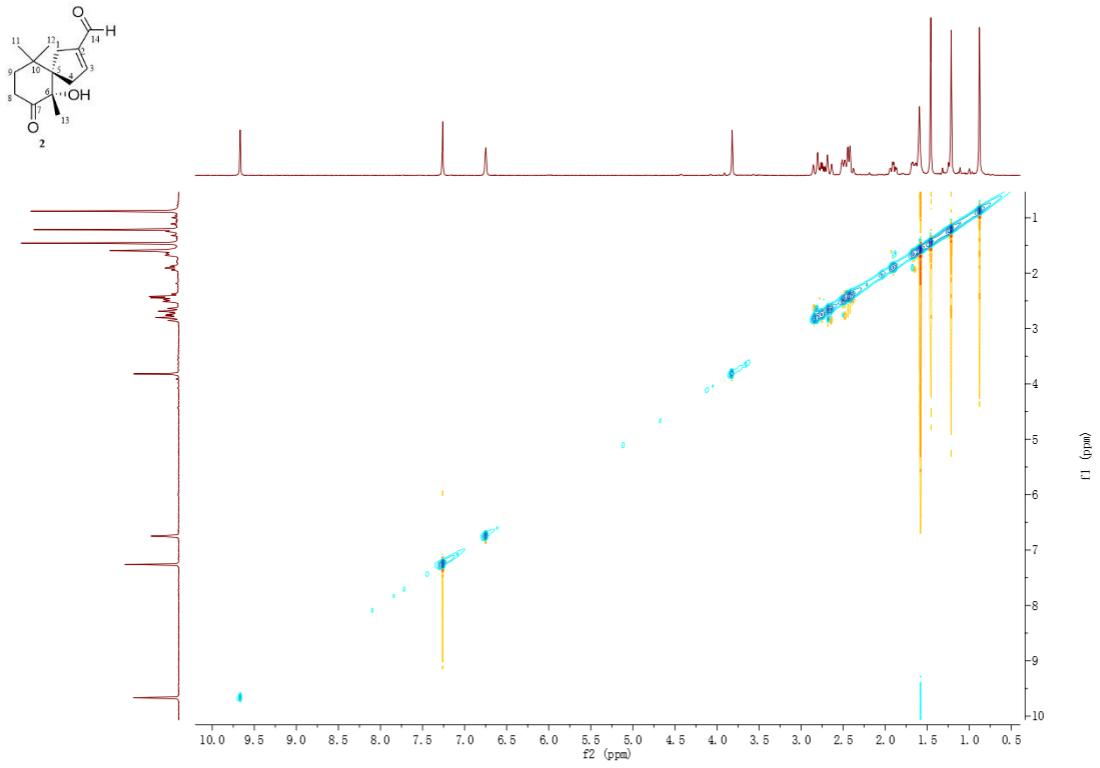

**Figure S17.** ROESY spectrum of **2**

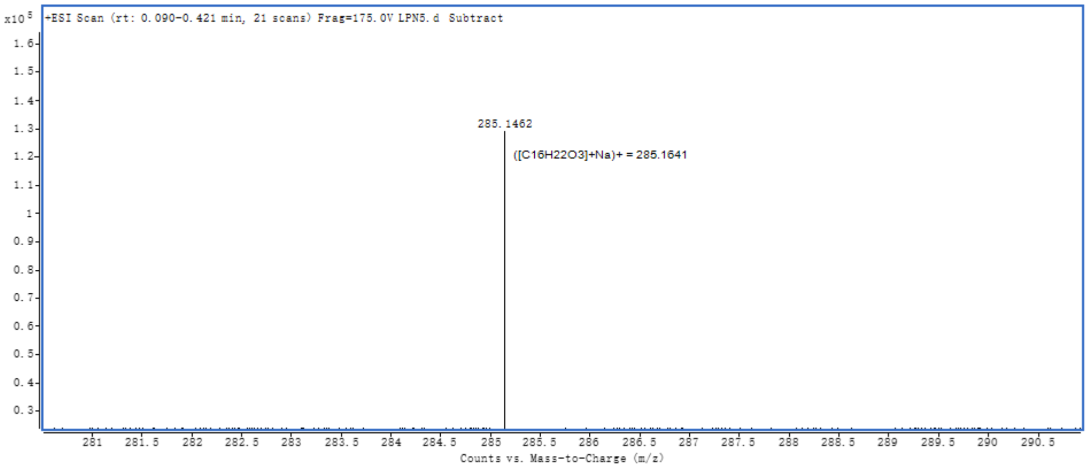


**Figure S18.** HR-ESI-MS spectrum of compound **3**

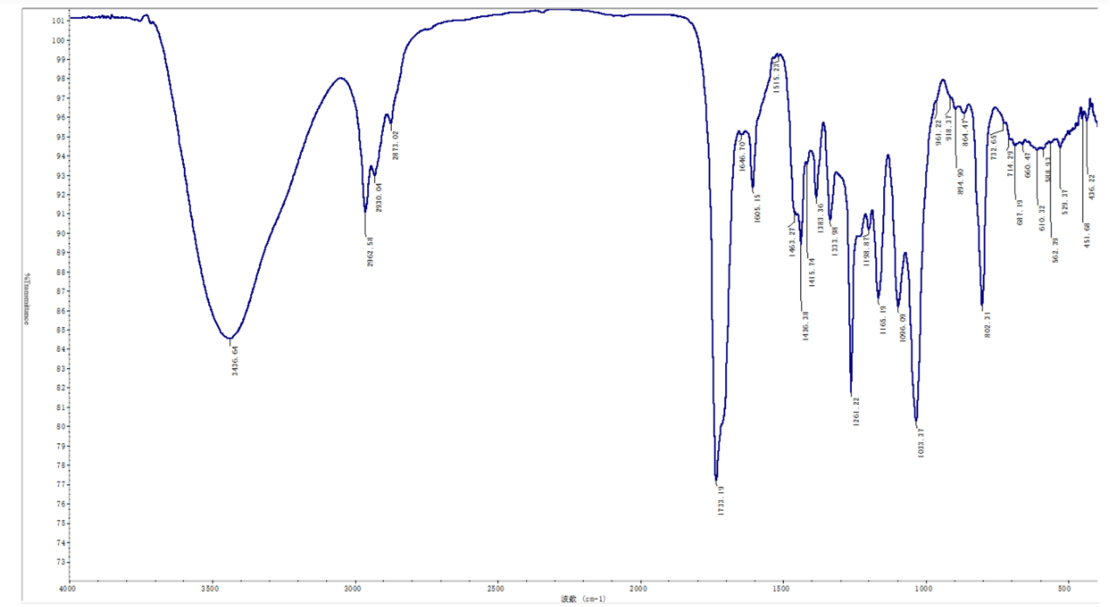

Sample Name: Lpn-5
KBr Pellets
Date: 15/11/2018 16:25:28 (GMT+08:00)
Instrument model: NICOLET iS10
Software version: OMNIC 9.8.372

Sample Scans: 16
Background Scans: 16
resolving Power: 4.000
Sampling gain: 1.0
Moving Mirror Speed: 0.4747
Diaphragm: 80.00

**Figure S19.** IR spectrum of compound **3**

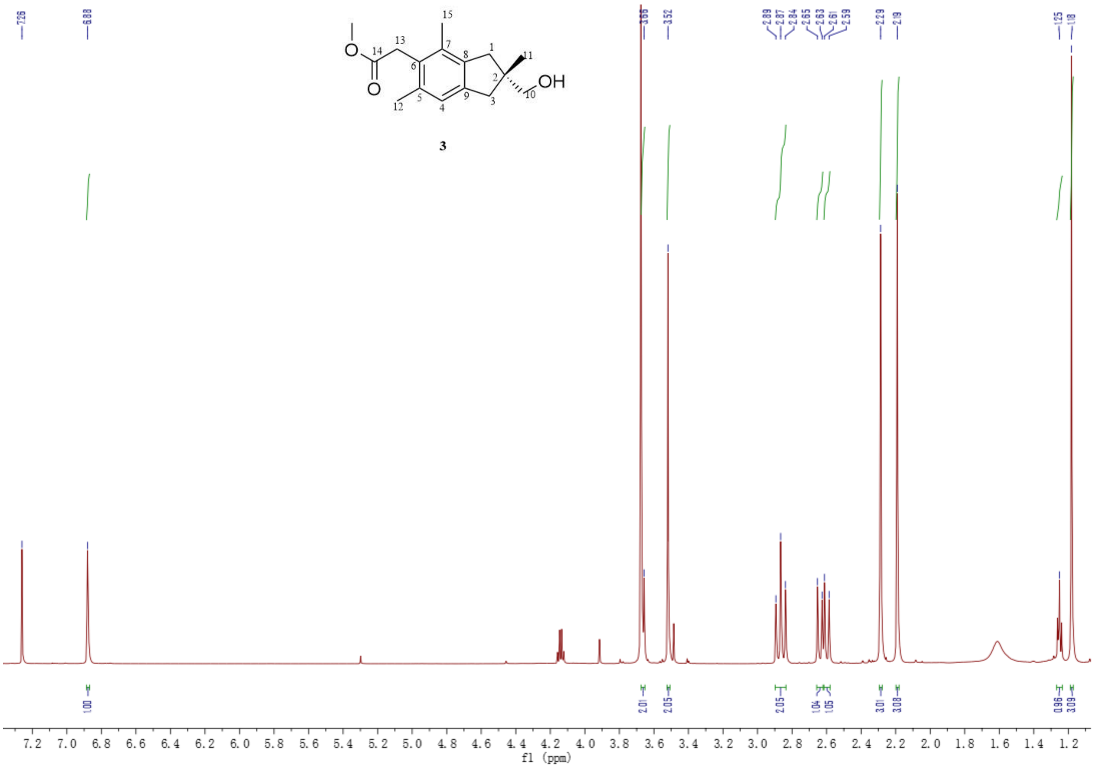

**Figure S20.** $^1$H NMR spectrum (600MHz, $CDCl_3$) of **3**

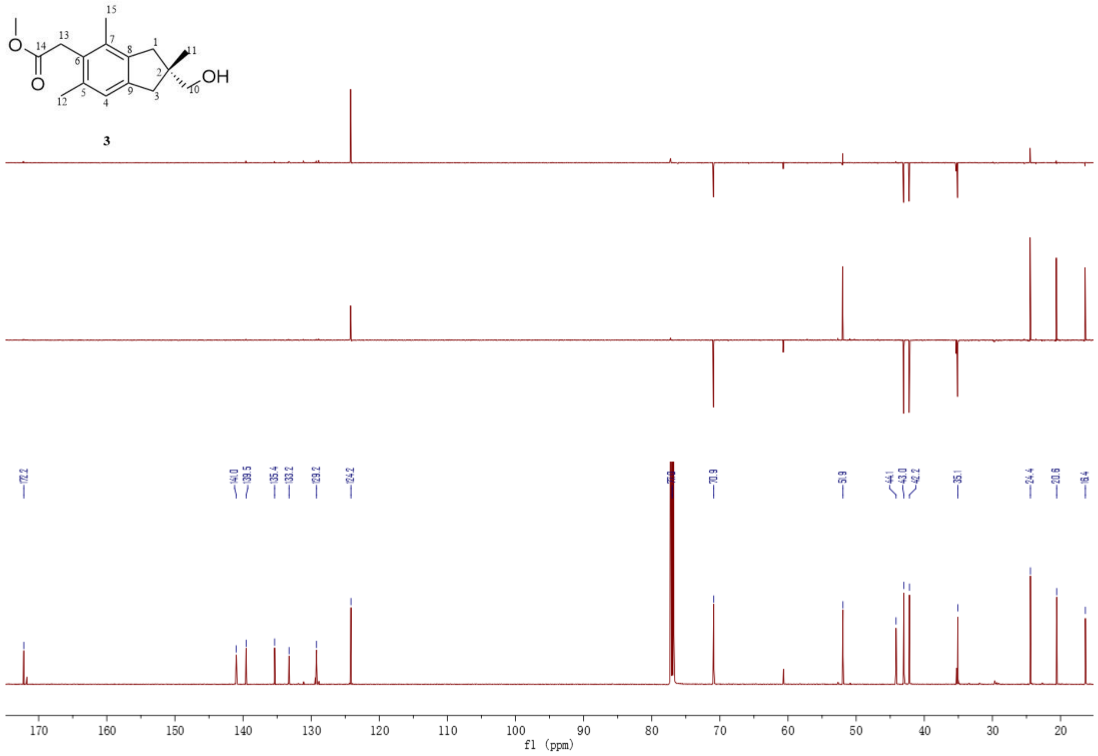


**Figure S21.** $^{13}$C and DEPT spectrum (150MHz, $CDCl_3$) of **3**

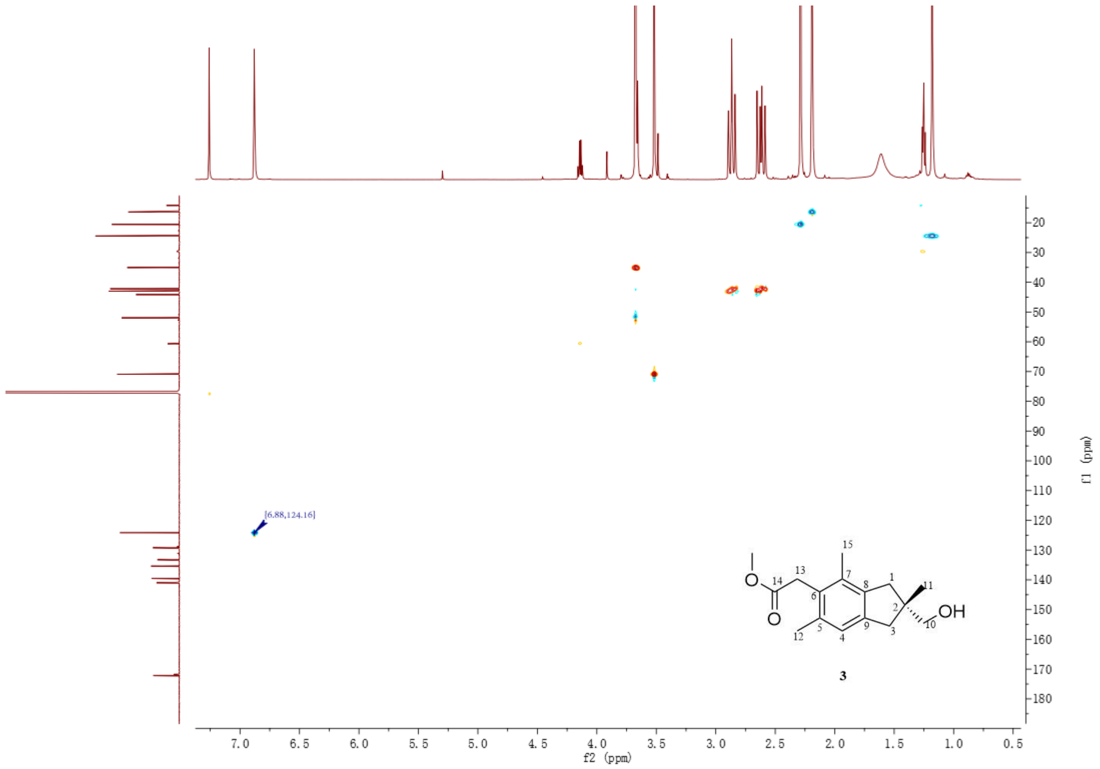


**Figure S22.** HSQC spectrum of **3**

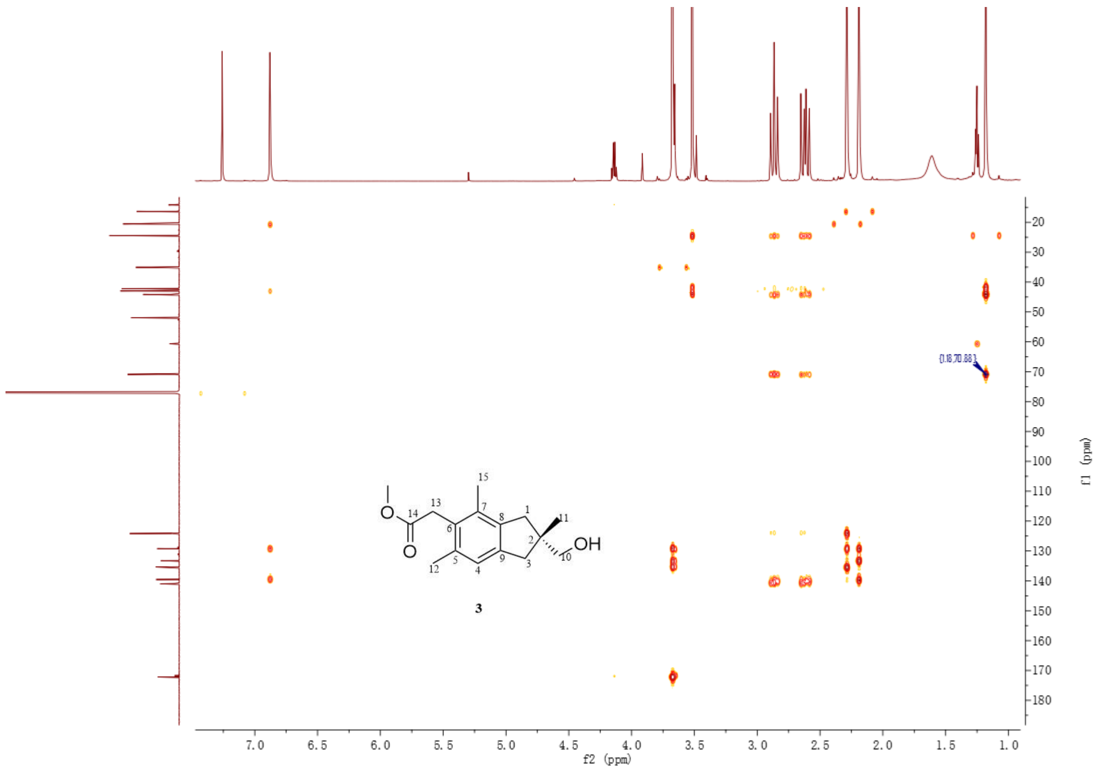

**Figure S23.** HMBC spectrum of **3**

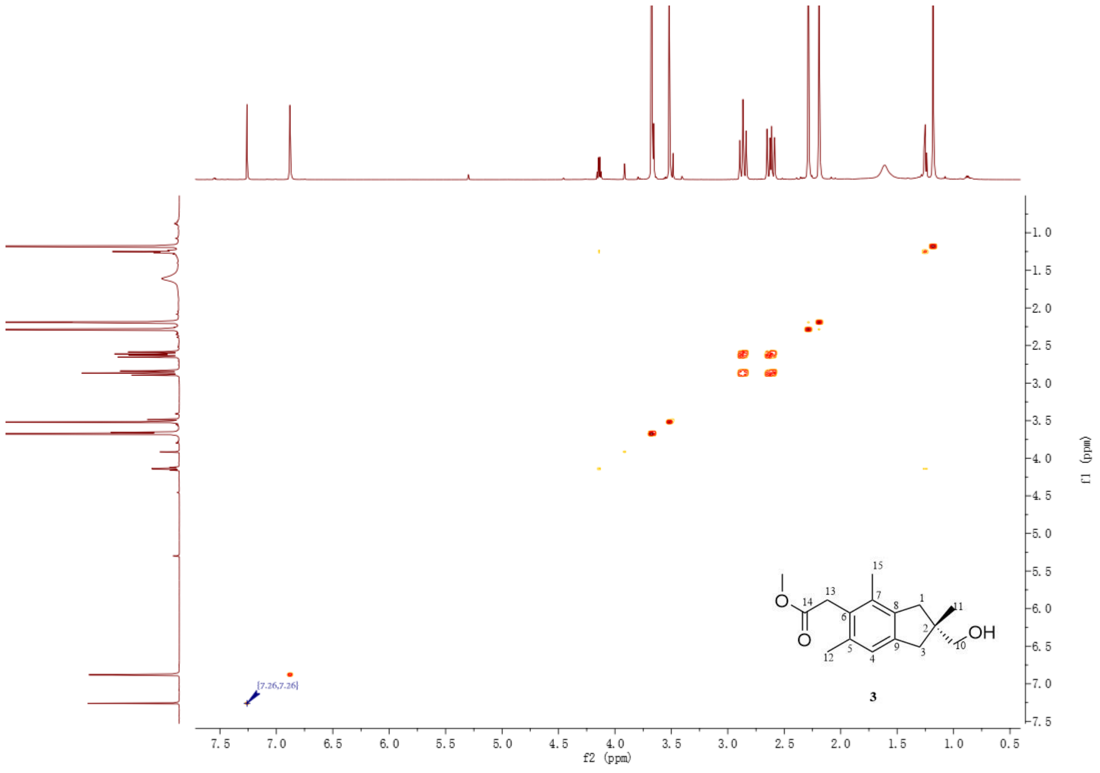

**Figure S24.** $^1H$-$^1H$ COSY spectrum of **3**

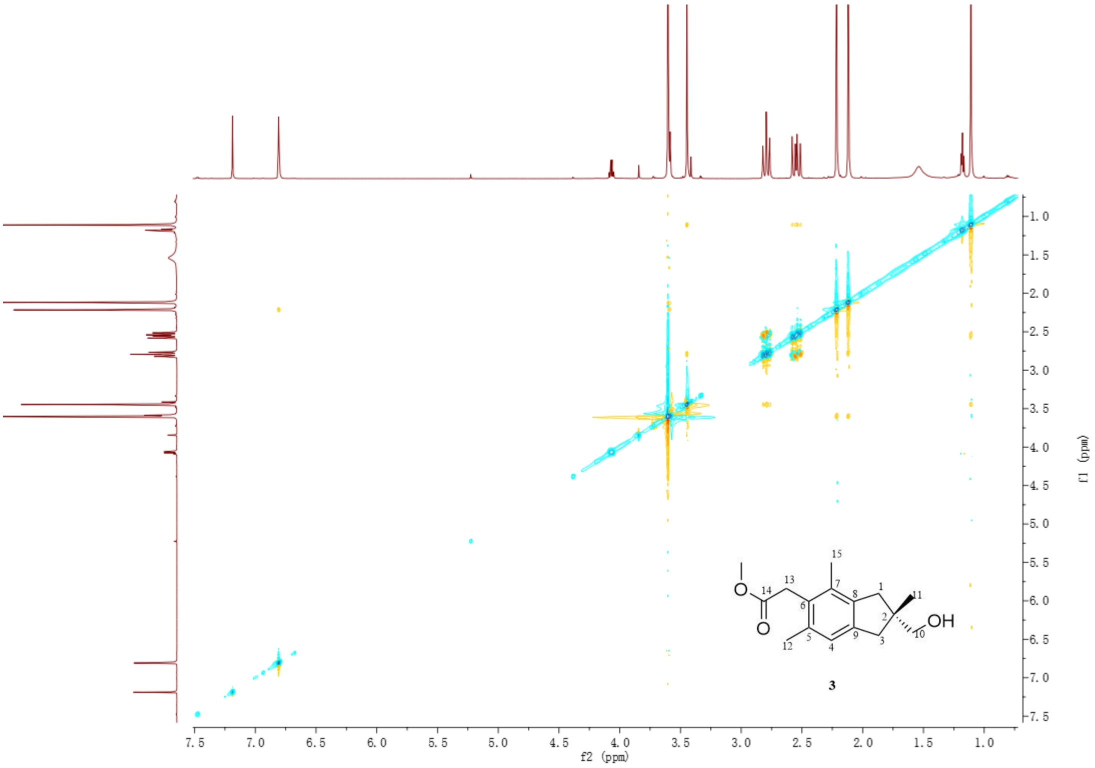


**Figure S25.** ROESY spectrum of **3**

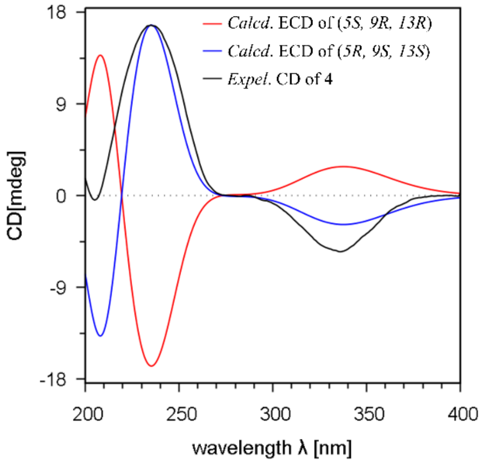


**Figure S26.** Experimental and calculated ECD spectra of compound **4**

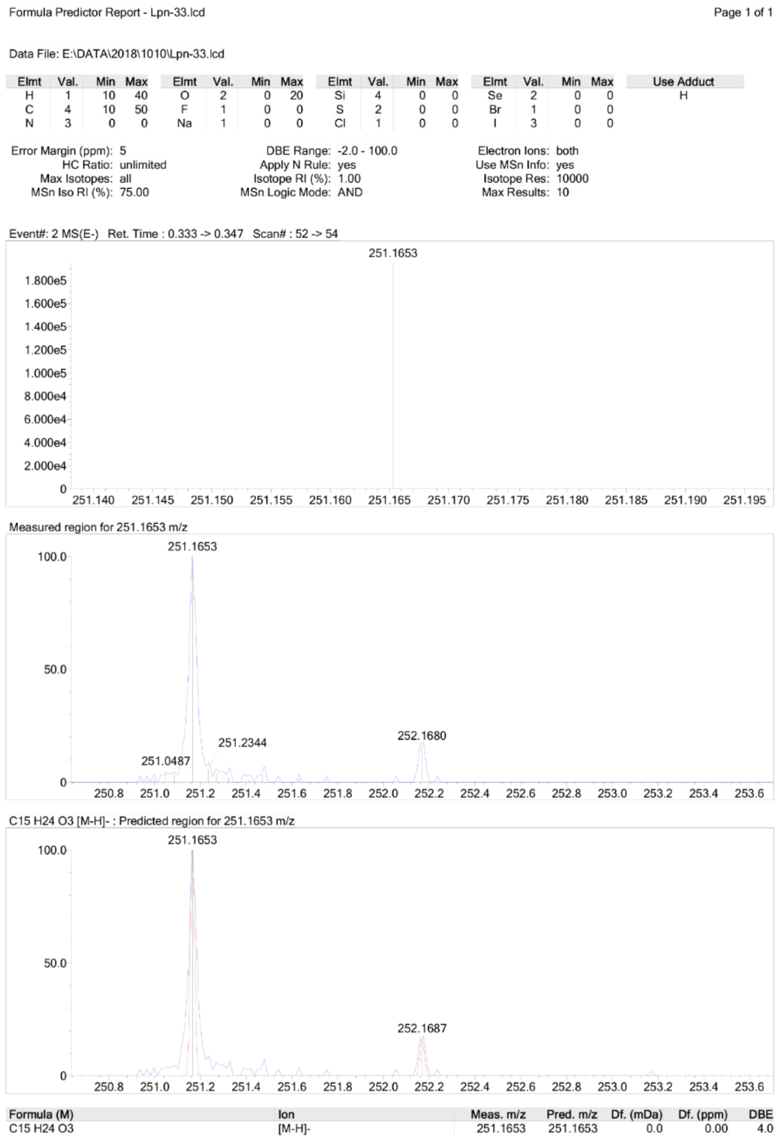

Formula Predictor Report - Lpn-33.lcd

Page 1 of 1

Data File: E:\DATA\2018\1010\Lpn-33.lcd

| Elmt | Val. | Min | Max | Elmt | Val. | Min | Max | Elmt | Val. | Min | Max | Elmt | Val. | Min | Max | Use Adduct |
|---|---|---|---|---|---|---|---|---|---|---|---|---|---|---|---|---|
| H | 1 | 10 | 40 | O | 2 | 0 | 20 | Si | 4 | 0 | 0 | Se | 2 | 0 | 0 | H |
| C | 4 | 10 | 50 | F | 1 | 0 | 0 | S | 2 | 0 | 0 | Br | 1 | 0 | 0 | |
| N | 3 | 0 | 0 | Na | 1 | 0 | 0 | Cl | 1 | 0 | 0 | I | 3 | 0 | 0 | |

| | | | | | |
|---|---|---|---|---|---|
| Error Margin (ppm): | 5 | DBE Range: | -2.0 - 100.0 | Electron Ions: | both |
| HC Ratio: | unlimited | Apply N Rule: | yes | Use MSn Info: | yes |
| Max Isotopes: | all | Isotope RI (%): | 1.00 | Isotope Res: | 10000 |
| MSn Iso RI (%): | 75.00 | MSn Logic Mode: | AND | Max Results: | 10 |

Event#: 2 MS(E-) Ret. Time : 0.333 -> 0.347 Scan# : 52 -> 54



Measured region for 251.1653 m/z



C15 H24 O3 [M-H]- : Predicted region for 251.1653 m/z



| Formula (M) | Ion | Meas. m/z | Pred. m/z | Df. (mDa) | Df. (ppm) | DBE |
|---|---|---|---|---|---|---|
| C15 H24 O3 | [M-H]- | 251.1653 | 251.1653 | 0.0 | 0.00 | 4.0 |

**Figure S27.** HR-ESI-MS spectrum of compound **4**

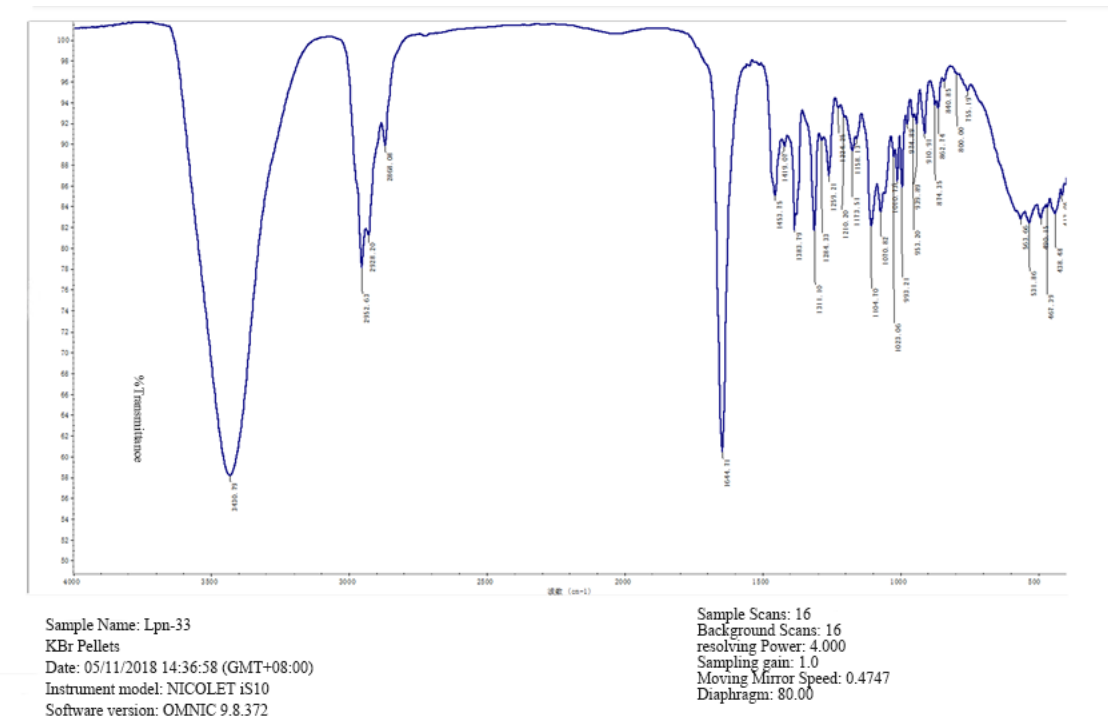


**Figure S28.** IR spectrum of compound **4**

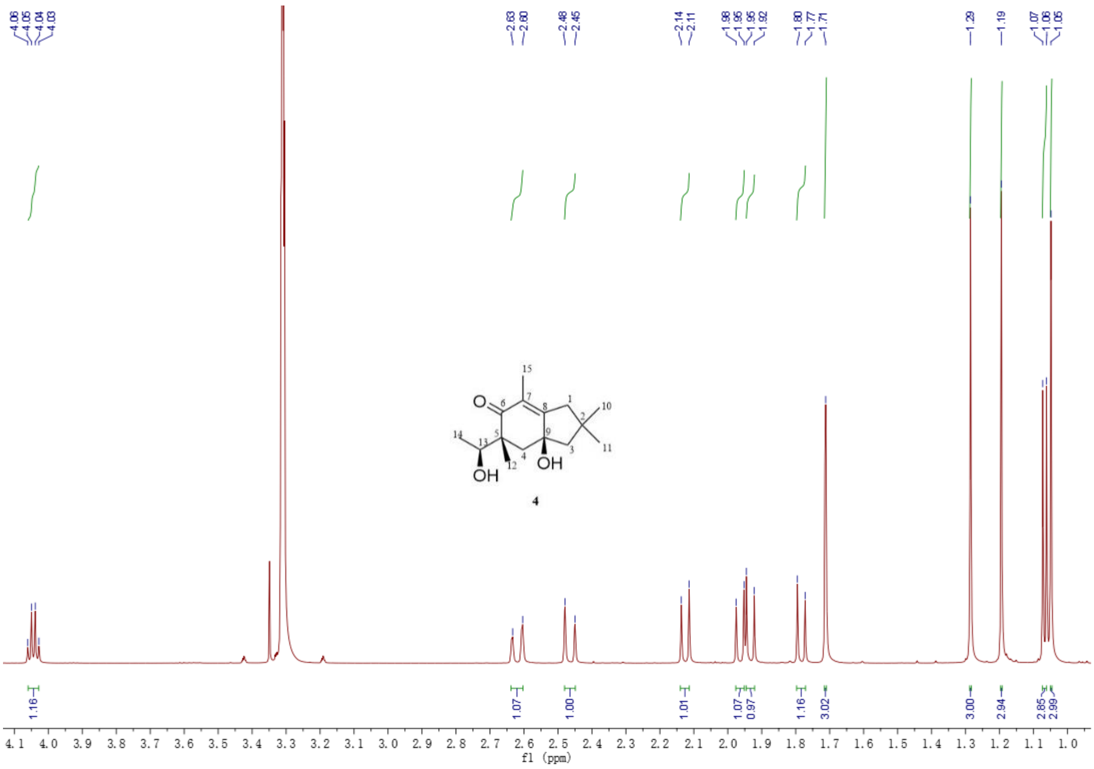


**Figure S29.** $^{1}$H NMR spectrum (600MHz, $CD_3OD$) of **4**

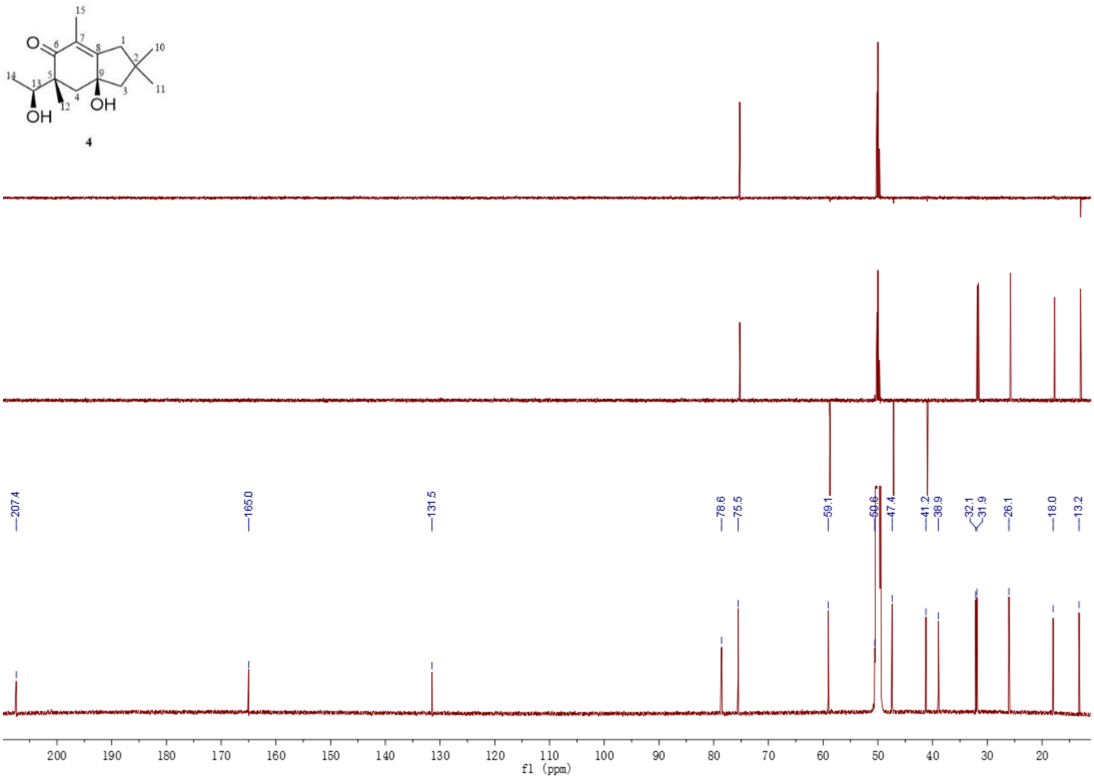


**Figure S30.** $^{13}C$ and DEPT spectrum (150MHz, $CD_3OD$) of **4**

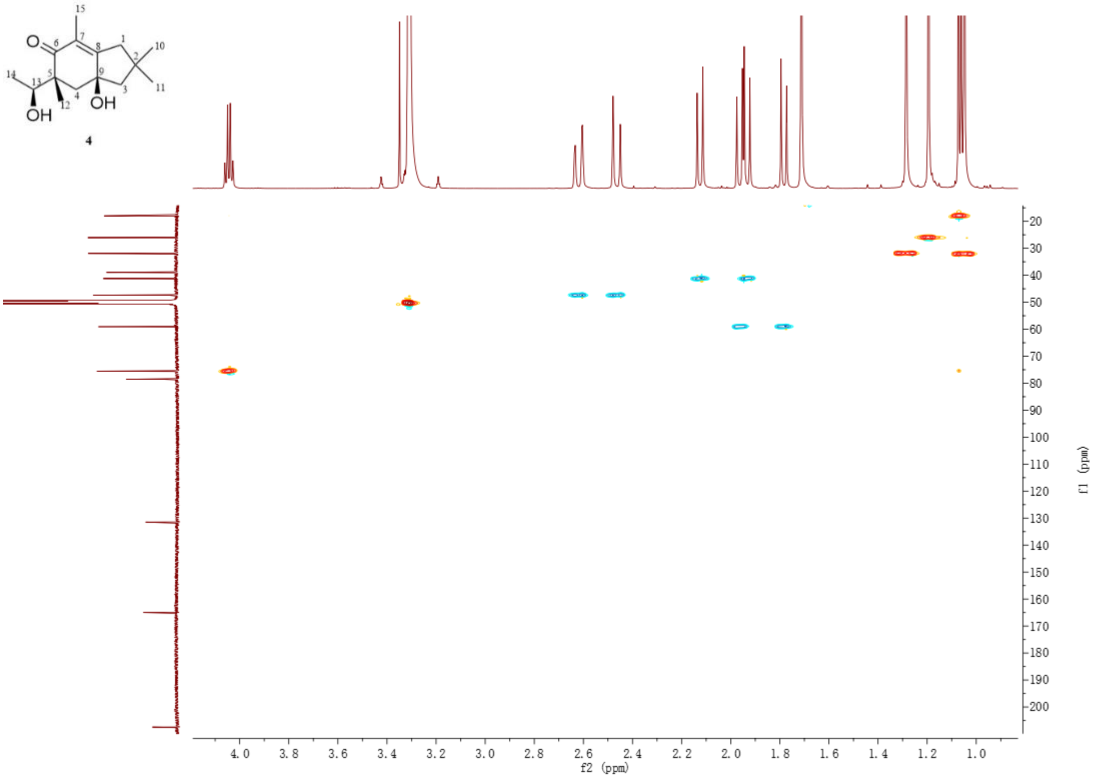


**Figure S31.** HSQC spectrum of **4**

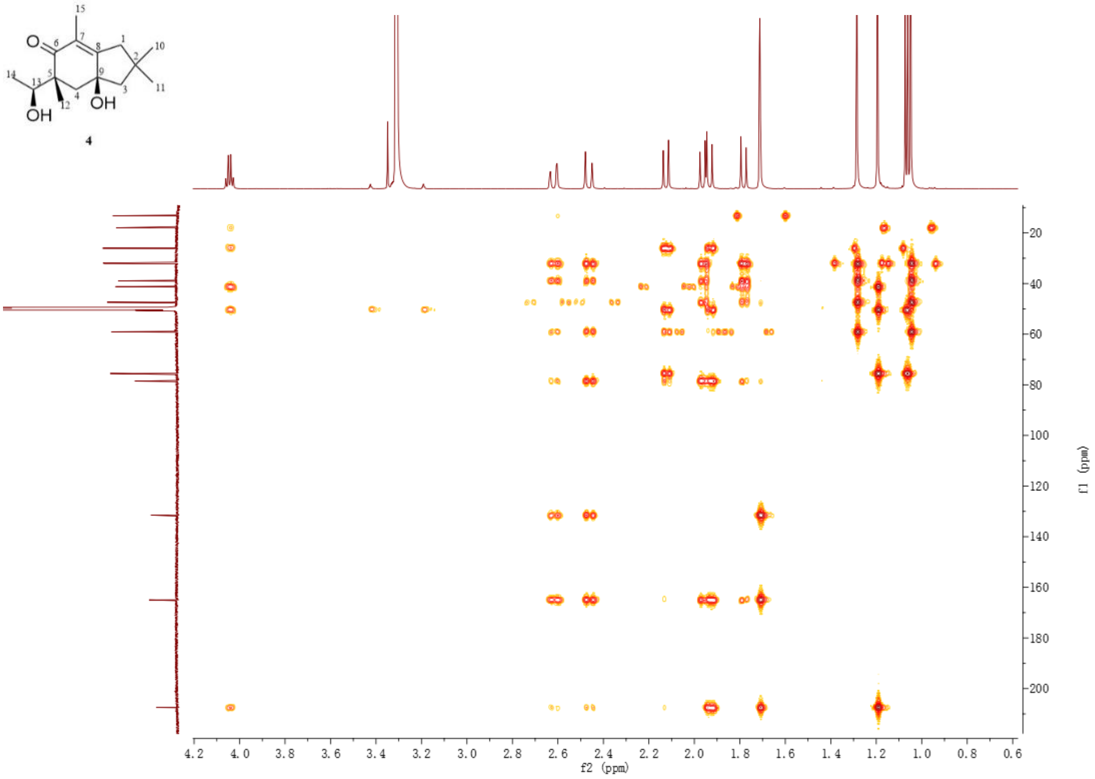


**Figure S32.** HMBC spectrum of **4**

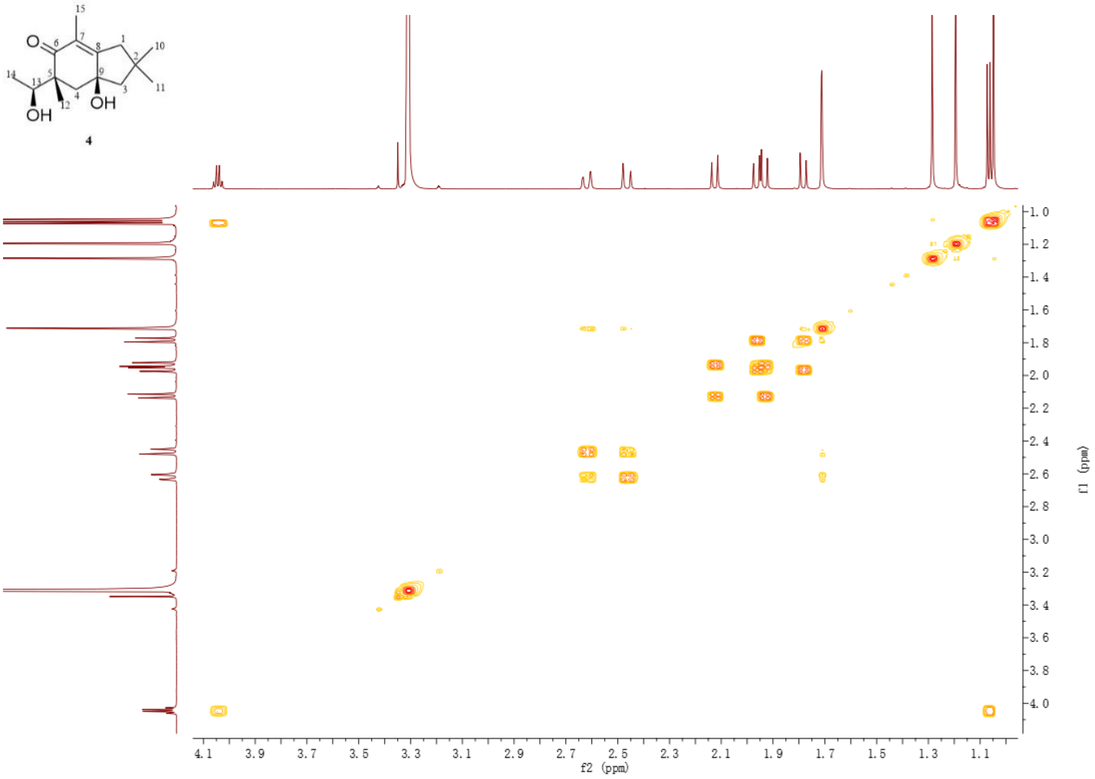

**Figure S33.** $^{1}$H-$^{1}$H COSY spectrum of **4**

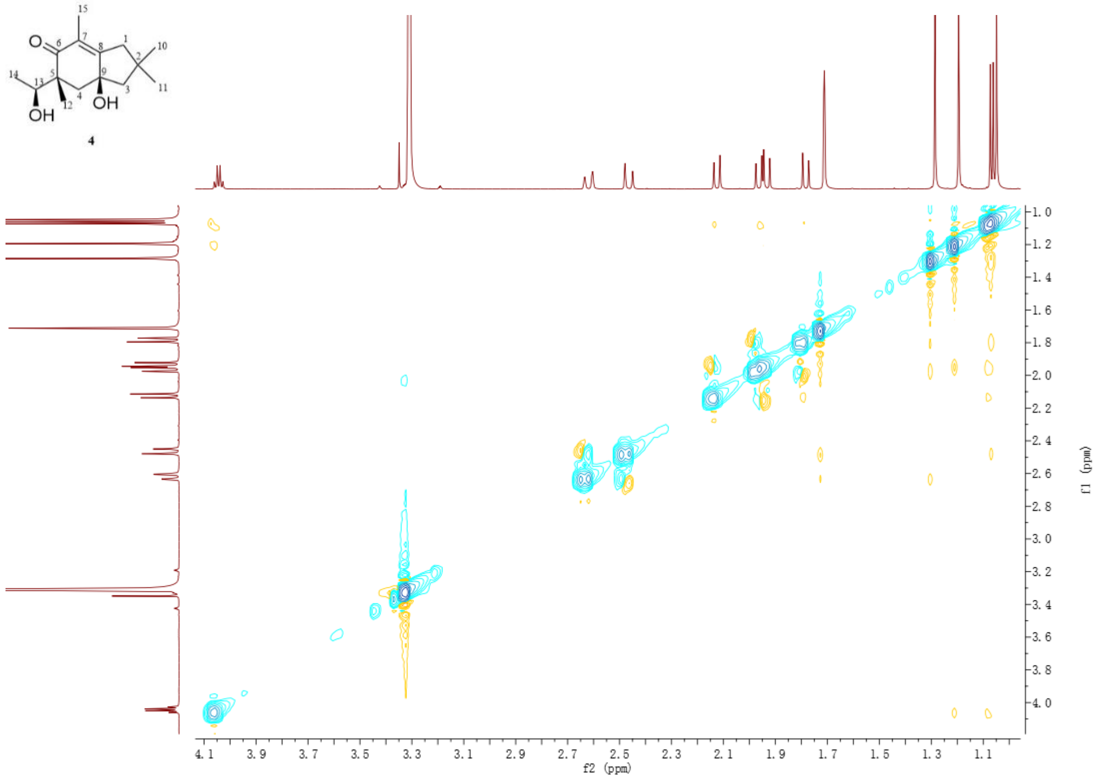


**Figure S34.** ROESY spectrum of **4**

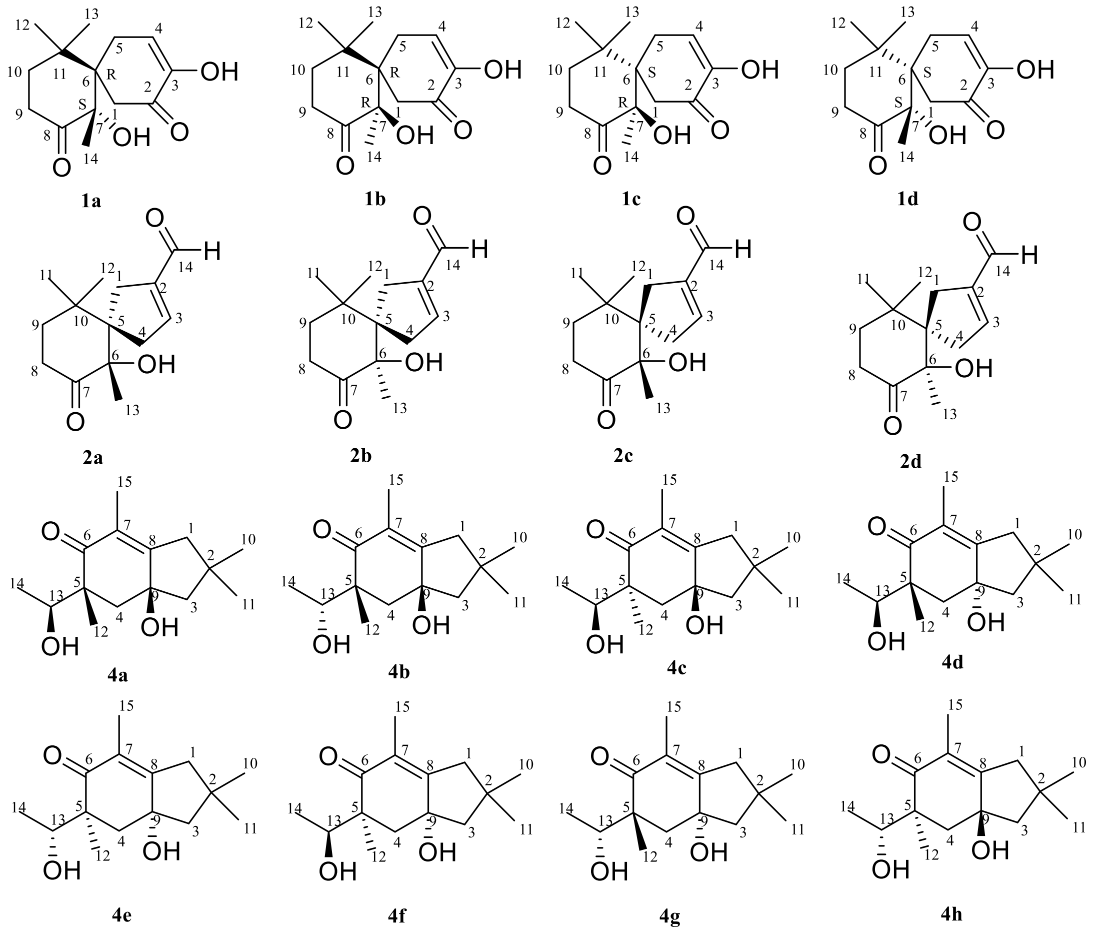


**Figure S35.** There are possible configurations of compounds **1**, **2** and **4**

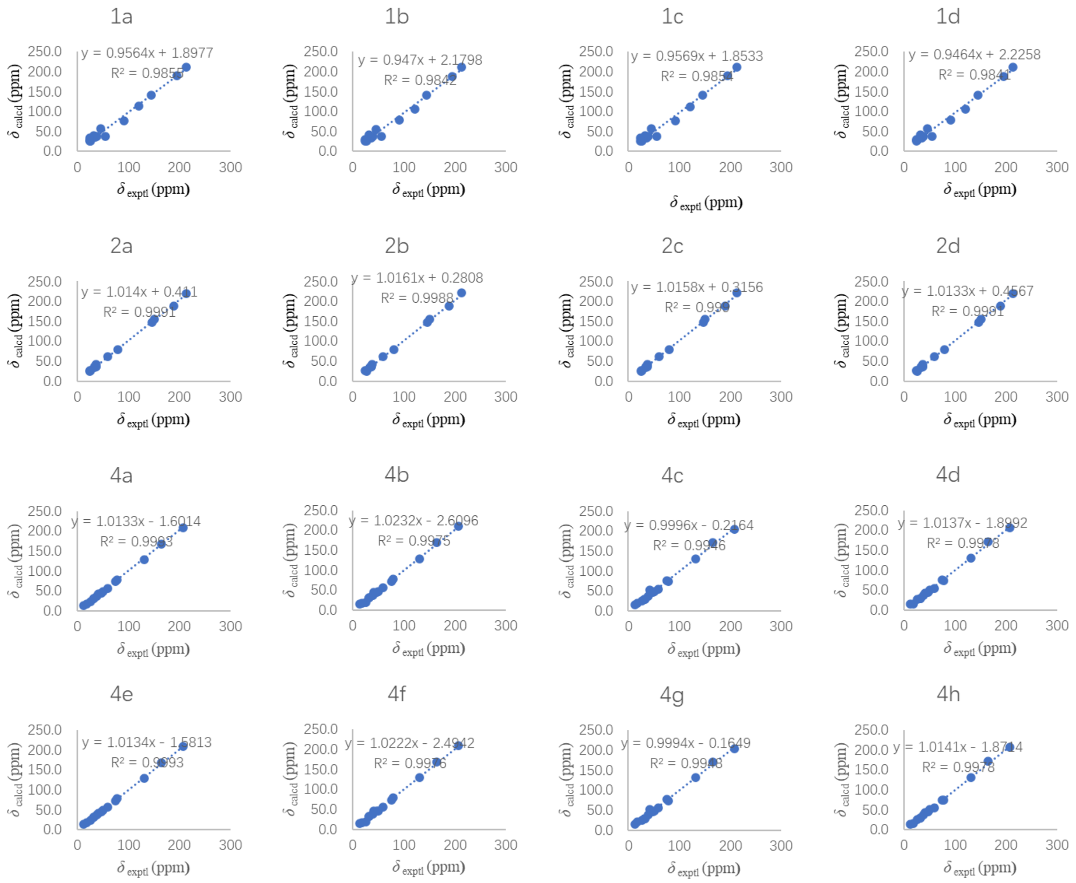


**Figure S36.** Linear correlation plots of calculate-experimental $^{13}C$ NMR chemical shift values for (6*R*,7*S*)-**1a**; (6*R*,7*R*)-**1b**; (6*S*,7*R*)-**1c**; (6*S*,7*S*)-**1d**; (5*S*,6*S*)-**2a**; (5*S*,6*R*)-**2b**; (5*R*,6*S*)-**2c**; (5*R*,6*R*)-**2d**, (5*R*,9*S*,13*S*)-**4a**; (5*R*,9*S*,13*R*)-**4b**; (5*S*,9*S*,13*S*)-**4c**; (5*R*,9*R*,13*S*)-**4d**; (5*S*,9*R*,13*R*)-**4e**; (5*S*,9*R*,13*S*)-**4f**; (5*R*,9*R*,13*R*)-**4g**; (5*S*,9*S*,13*R*)-**4h**

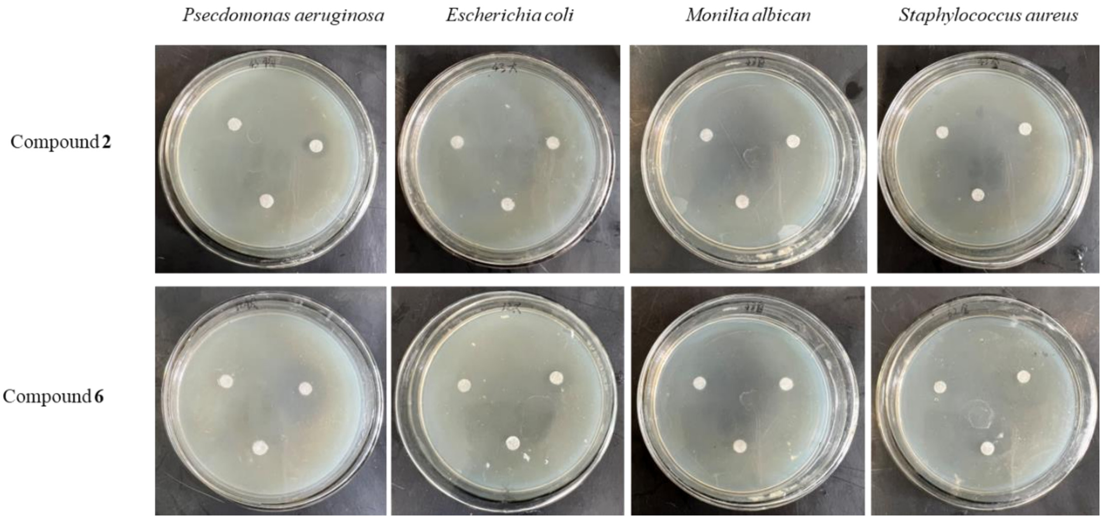


**Figure S37.** Antibacterial test of compounds **2** and **6**

Calculation of optical rotation of compound **2**:

The conformation search found that compound **2** (5*S*,6*S*) has three conformations, among which the Boltzmann distribution ratio and OR value of each conformation are as follows:

**Table S4.** The Boltzmann distribution proportion of its dominant conformation of the compound **2** (5*S*,6*S*)

| Comformation | Weight (%) | OR |
|---|---|---|
| 1 | 99.43 | -9.79 |
| 2 | 0.44 | -8.32 |
| 3 | 0.12 | -8.48 |

Calculation of optical rotation of compound **3**:

Through conformation search, it is found that there are 124 conformations with the energy threshold of compound **3** (*S*) within 3.0 kcal/mol, and the Boltzmann distribution proportion of its dominant conformation is shown in table S4：

**Table S5.** Compound **3** (*S*) within 3.0 kcal/mol, and the Boltzmann distribution proportion of its dominant conformation

| Comfo-rmation | Weight (%) | OR | Comfo-rmation | Weight (%) | OR | Comfo-rmation | Weight (%) | OR |
|---|---|---|---|---|---|---|---|---|
| 1 | 1.56 | -7.21 | 62 | 1.07 | 134.05 | 101 | 1.16 | 149.56 |
| 3 | 1.2 | -69.09 | 68 | 1.08 | 68.84 | 102 | 1.41 | 17.51 |

| | | | | | | | | |
|---|---|---|---|---|---|---|---|---|
| 4 | 1.43 | -1.8 | 69 | 2 | -3.08 | 103 | 1.65 | 22.33 |
| 5 | 1.13 | 81.11 | 70 | 1.02 | 83.17 | 104 | 1.23 | 87.58 |
| 8 | 1.59 | -6.89 | 72 | 1.63 | -91.08 | 105 | 2.18 | 70.96 |
| 17 | 1.16 | 82.37 | 73 | 1.54 | 82.4 | 106 | 1.69 | -58.1 |
| 18 | 1.43 | 8.31 | 75 | 1.17 | -248.81 | 108 | 1.6 | -41.23 |
| 20 | 1.43 | 31.95 | 76 | 1.11 | 158.47 | 109 | 1.16 | -266.44 |
| 21 | 1.54 | 188.75 | 78 | 0.99 | 60.23 | 110 | 1.39 | -183.21 |
| 24 | 1.24 | -64.94 | 79 | 1.04 | 42.8 | 111 | 1.62 | 25.44 |
| 27 | 1.76 | 84.65 | 81 | 2.02 | -13.43 | 112 | 1.25 | -76.34 |
| 31 | 1.53 | 171.84 | 84 | 1.12 | 155.41 | 113 | 1.43 | -61.62 |
| 37 | 1.49 | 107.52 | 85 | 1.05 | -263.5 | 114 | 1.25 | 97.81 |
| 41 | 1.45 | 15.27 | 89 | 2.16 | 3.49 | 115 | 1.33 | -230.92 |
| 43 | 1.02 | 140.03 | 90 | 2.26 | 166.56 | 116 | 1.42 | 27.8 |
| 44 | 1.02 | 44.13 | 91 | 1.06 | 133.4 | 117 | 1.41 | -164.63 |
| 50 | 1.04 | 73.11 | 93 | 1.03 | 67.55 | 118 | 1.58 | -37.89 |
| 52 | 2.11 | -1.43 | 94 | 1.02 | 40.68 | 119 | 1.31 | -247.35 |
| 53 | 1.17 | -257.65 | 95 | 2.22 | 46.27 | 120 | 1.43 | -125.95 |
| 55 | 1.51 | 113.57 | 96 | 1.56 | -9.98 | 121 | 1.39 | -52.4 |
| 56 | 1.77 | 79.7 | 98 | 2.07 | 78.53 | 122 | 1.42 | -121.11 |
| 57 | 1.27 | 70.01 | 99 | 1.65 | -85.72 | 123 | 1.24 | 101.05 |
| 60 | 1 | 31.15 | 100 | 2.18 | -5.19 | 124 | 1.41 | -120.26 |
| 61 | 1.17 | -253.2 | | | | | | |

The calculation results show that the calculated optical rotation value of **3** (*S*) is +3.01, while the experimental optical rotation value (as shown in the figure S37) is -2.92. Therefore, it is judged that the absolute configuration of compound **3** is *R*.

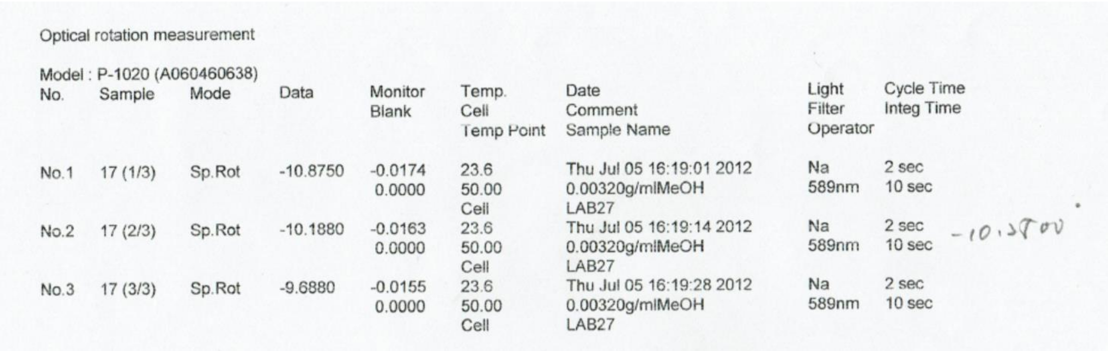
Optical rotation measurement

Model : P-1020 (A060460638)

| No. | Sample | Mode | Data | Monitor / Blank | Temp. / Cell / Temp Point | Date / Comment / Sample Name | Light / Filter / Operator | Cycle Time / Integ Time |
|---|---|---|---|---|---|---|---|---|
| No.1 | 17 (1/3) | Sp.Rot | -10.8750 | -0.0174 / 0.0000 | 23.6 / 50.00 / Cell | Thu Jul 05 16:19:01 2012 / 0.00320g/mlMeOH / LAB27 | Na / 589nm | 2 sec / 10 sec |
| No.2 | 17 (2/3) | Sp.Rot | -10.1880 | -0.0163 / 0.0000 | 23.6 / 50.00 / Cell | Thu Jul 05 16:19:14 2012 / 0.00320g/mlMeOH / LAB27 | Na / 589nm | 2 sec / 10 sec |
| No.3 | 17 (3/3) | Sp.Rot | -9.6880 | -0.0155 / 0.0000 | 23.6 / 50.00 / Cell | Thu Jul 05 16:19:28 2012 / 0.00320g/mlMeOH / LAB27 | Na / 589nm | 2 sec / 10 sec |

**Figure S38.** Experimental optical rotation of compound **2**

| | | | | | | |
|---|---|---|---|---|---|---|
| 17 (1/3) | Specific O.R. | -3.310 | 24.7 | 0 | Mon Sep 03 18:15:15 2018 | 0.00290g/mL MeOH LPN-5 |
| 17 (2/3) | Specific O.R. | -2.138 | 24.8 | 0 | Mon Sep 03 18:15:21 2018 | 0.00290g/mL MeOH LPN-5 |
| 17 (3/3) | Specific O.R. | -3.310 | 24.8 | 0 | Mon Sep 03 18:15:26 2018 | 0.00290g/mL MeOH LPN-5 |

**Figure S39.** Experimental optical rotation of compound **3**
